# Silver Nanoparticles as Antimicrobial Agents in Veterinary Medicine: Current Applications and Future Perspectives

**DOI:** 10.3390/nano15030202

**Published:** 2025-01-27

**Authors:** Thibault Frippiat, Tatiana Art, Catherine Delguste

**Affiliations:** 1 Fundamental and Applied Research for Animals & Health (FARAH), Faculty of Veterinary Medicine, University of Liège, 4000 Liège, Belgium; 2 Sportpaardenarts—Equine Sports Medicine, 1250AD Laren, The Netherlands

**Keywords:** silver, nanoparticles, animals, veterinary, antibacterial agents, antiviral agents, antifungal agents

## Abstract

Silver nanoparticles (AgNPs) have gained significant attention in veterinary medicine due to their antimicrobial properties and potential therapeutic applications. Silver has long been recognized for its ability to combat a wide range of pathogens, and when engineered at the nanoscale, silver’s surface area and reactivity are greatly enhanced, making it highly effective against bacteria, viruses, and fungi. This narrative review aimed to summarize the evidence on the antimicrobial properties of AgNPs and their current and potential clinical applications in veterinary medicine. The antimicrobial action of AgNPs involves several mechanisms, including, among others, the release of silver ions, disruption of cell membranes and envelopes, induction of oxidative stress, inhibition of pathogens’ replication, and DNA damage. Their size, shape, surface charge, and concentration influence their efficacy against bacteria, viruses, and fungi. As a result, the use of AgNPs has been explored in animals for infection prevention and treatment in some areas, such as wound care, coating of surgical implants, animal reproduction, and airway infections. They have also shown promise in preventing biofilm formation, a major challenge in treating chronic bacterial infections. Additionally, AgNPs have been studied for their potential use in animal feed as a supplement to enhance animal health and growth. Research suggested that AgNPs could stimulate immune responses and improve the gut microbiota of livestock, potentially reducing the need for antibiotics in animal husbandry. Despite their promising applications, further research is necessary to fully understand the safety, efficacy, and long-term effects of AgNPs on animals, humans, and the environment.

## 1. Introduction

Silver, a transition metal with atomic number 47, is known for its remarkable properties. Its nuclear structure features a single electron in the outermost shell, contributing to its high reactivity, particularly with sulfur and halogens. Silver exists in various oxidation states, with the most common being elemental silver (Ag^0^) and silver ion (Ag⁺). The neutral form, Ag⁰, is the metallic state of silver, characterized by its high electrical and thermal conductivity, malleability, and corrosion resistance. This form of silver is widely used in coins, jewelry, and industrial applications. In contrast, Ag⁺, formed when silver loses an electron, is typically encountered in compounds such as silver nitrate and silver chloride. It is highly reactive, capable of forming complexes with various ligands, and exhibits antimicrobial properties, making it useful in medical and industrial applications. It is worth noting that while silver nitrate is highly soluble in water, silver chloride is insoluble.

Nanoparticles are particles with dimensions typically ranging from 1 to 100 nanometers (nm). Due to their small size, they exhibit unique physical and chemical properties compared to their bulk counterparts, such as increased surface area-to-volume ratio and enhanced reactivity. These properties make nanoparticles valuable in various fields, including medicine, electronics, and environmental science. Nanoparticles can be classified into different types based on composition and morphology, including metallic, polymeric, and carbon-based forms. Common shapes include spherical, rod-like, and tube-like structures, each affecting their interaction with their surrounding environments.

The history of silver nanoparticles (AgNPs) in medicine and veterinary medicine is marked by a significant evolution in their applications, primarily driven by their antimicrobial properties. Silver has been recognized for its therapeutic benefits for centuries, with its use dating back to ancient civilizations, where it was employed for wound healing and as a preservative in food and water [1,2]. The advent of nanotechnology in recent years has revolutionized the utilization of silver. The increased efficacy compared to bulk silver has led to a resurgence of interest in AgNPs as potent antimicrobial agents in both human and veterinary medicine [3]. Their effectiveness against a broad spectrum of pathogens, including bacteria, viruses, and fungi, positions them as a promising alternative to antibiotics, especially in the context of rising antibiotic resistance in veterinary settings [4,5,6].

The antimicrobial activity of AgNPs is primarily driven by their oxidation state [7]. Silver remains stable in water but requires an oxidizing agent, such as oxygen or hydrogen peroxide (H_2_O_2_), to undergo oxidative dissolution, a process that releases Ag⁺ via an oxidative mechanism [8,9]. This release leads to the generation of reactive oxygen species (ROS) within cells, further promoting AgNP dissolution [10]. This oxidation of Ag^0^ is a key mechanism underlying the antimicrobial activity of AgNPs, as the resulting Ag⁺ interacts with microbial components. The redox potential of Ag^0^ is influenced by physical factors, including AgNPs’ size and surface coatings. The high surface area-to-volume ratio of AgNPs significantly impacts their redox properties, with smaller particles exhibiting higher surface energy, which lowers the activation energy required for oxidation and enhances the conversion of Ag^0^ to Ag^+^ [11]. Additionally, environmental factors, such as pH, halide ions, reducing sugars, cysteine, and natural organic matter, also play a role by modifying the redox potential, altering the surface chemistry, and affecting the accessibility of reactive sites [8,12,13].

**Figure 1:**
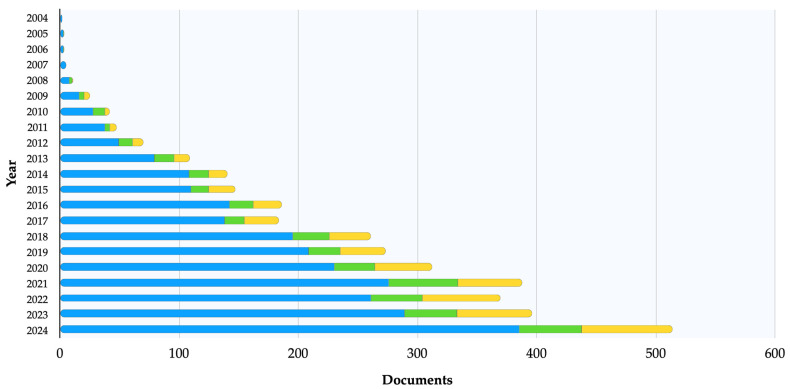
Document counts (*n* = 3492) on the antimicrobial activity of silver nanoparticles in animals from years 2004 to 2024, obtained using the keywords (silver AND nanoparticles) AND (veterinary OR animal) AND one of the following pathogen-specific terms: for bacteria (blue) using (bacteria OR antibacterial OR bactericidal), for viruses (green) using (virus OR antiviral OR virucidal), or for fungi (yellow) using (fungus OR fungi OR antifungal OR fungicidal). Data were retrieved from Scopus Search (https://www.scopus.com/search/form.uri (accessed on 16 January 2025)). Notably, antibacterial activity has been investigated more extensively than antiviral and antifungal activities.

The use of AgNPs as antimicrobials in animals has gained increasing interest in recent years (Figure 1). This narrative review aimed to summarize the evidence of AgNPs’ actual applications in veterinary medicine and identify future perspectives in clinical use and research.

## 2. Synthesis of Silver Nanoparticles

**Figure 2:**
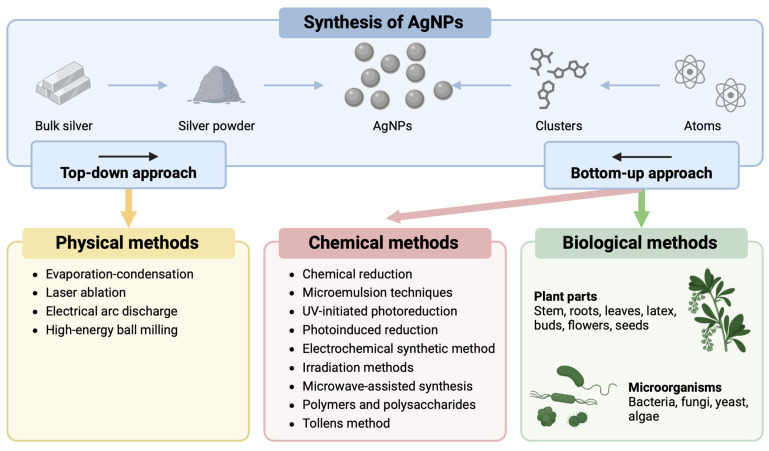
Different approaches for the synthesis of silver nanoparticles (AgNPs), according to [14].

Various methods can be used to synthesize AgNPs, including chemical, physical, and biological synthesis, which are mainly divided into two processes: top-down and bottom-up [14] (Figure 2). The top-down approach—essentially the physical methods— involves the creation of AgNPs from bulk silver by applying various physical forces. This approach allows for the acquisition of pure AgNPs without chemical additives and with a uniform size distribution [15]. The bottom-up approach—mainly the chemical and biological methods—involves the assembly of complex clusters to form AgNPs from molecular components through nucleation and growth processes. While the chemical methods allow for the rapid production of AgNPs, the use of chemical additives could restrict their medical applications [16]. Biological methods have emerged as promising alternatives to address these limitations. This economical, environmentally friendly, simple, and reliable approach uses macromolecules from bacteria, fungi, and algae and organic components from plant extracts [17].

### 2.1. Physical Synthesis

Physically synthesized AgNPs tend to have a high uniform particle size distribution and higher purity. However, a drawback of this method is the absence of chemical additives, such as stabilizers or capping agents, which, while potentially harmful to organisms or the environment, help prevent particle agglomeration [18]. Additionally, these processes often require sophisticated equipment and significant external energy input.

Different energy sources, including mechanical, thermal, light, and electrical, may be used to reduce silver particle size during the physical synthesis of AgNPs. High-energy ball milling is a mechanical method, in which intense mechanical forces, applied through the collision of grinding media within a ball mill, break down bulk silver into fine AgNPs through repeated fracturing and cold-welding processes [19]. The arc-discharge method involves generating high-temperature plasma between two electrodes in an inert gas atmosphere, where the intense heat vaporizes the silver, and the vapor subsequently condenses to form AgNPs [20]. Laser ablation synthesis involves using a high-powered laser to irradiate a silver target submerged in a liquid, causing rapid material vaporization and subsequent condensation into AgNPs in the surrounding medium [21]. Finally, physical vapor deposition involves the evaporation of silver in a vacuum environment, where the vaporized material condenses onto a substrate to form AgNPs through processes like sputtering or thermal evaporation [22]. Physical synthesis allows for the production of AgNPs on a large scale, but they tend to aggregate and form larger particles, affecting their application potential and properties. Aggregated AgNPs may hinder their attachment to bacteria, potentially resulting in reduced antibacterial effectiveness [23]. Notably, larger-sized AgNPs demonstrated a greater tendency to aggregate compared to smaller ones [24].

### 2.2. Chemical Synthesis

The chemical synthesis of AgNPs is one of the most widely employed methods due to its simplicity, versatility, and ability to control nanoparticle size and morphology. This method involves the reduction of silver salts, most commonly silver nitrate, to form metallic AgNPs. This reduction process is typically facilitated by a chemical reducing agent, which donates electrons to Ag^+^, reducing them to Ag^0^, and a stabilizing agent to prevent aggregation of the nanoparticles during and after their formation. Sodium borohydride is the most commonly used reducing agent due to its high reduction potential, allowing for rapid and controlled reduction of Ag^+^ at relatively low temperatures [25]. Other frequent reducing agents include sodium citrate, a milder agent facilitating a more gradual reduction process that can influence the nanoparticles’ shape and size [26], and ascorbic acid and glucose, particularly used in environmentally friendly or “green” synthesis routes [27,28]. The use of toxic chemicals in the chemical synthesis of AgNPs makes the resulting particles less suitable for medical, cosmetic, or food applications [29].

Stabilizers are often employed to prevent reaggregation and maintain stable suspensions [30]. Two common stabilizers are typically used. Citrates—low-molecular-weight ions—are the most commonly used reducing agent for stabilizing AgNPs [31]. They impart a negative surface charge to AgNPs, protecting them through electrostatic repulsion. Polyethylene glycol and polyvinylpyrrolidone—hydrophilic, neutral, high-molecular-weight polymers—are other popular coatings, particularly suitable for biomedical applications due to their biocompatibility and the enhanced stability they provide to AgNPs [32]. They provide protection by concealing surface charges and creating steric hindrance, which prevents interactions with other AgNPs, reducing aggregation and ensuring high stability [33,34]. The natural polymers not only stabilize the nanoparticles but can also impart additional functionality, such as biocompatibility [35]. These chemical methods provide reasonable control over AgNPs’ size but often require high-energy inputs, involve toxic chemicals, necessitate controlled temperature and pressure, and lead to environmental concerns and high costs [36].

### 2.3. Biological Synthesis

Biological synthesis has emerged as an eco-friendly and more cost-effective alternative in the field of “green chemistry” [3,17,36]. This method is advantageous as it can be performed at room temperature and pressure without the need for organic solvents. Biological approaches are particularly attractive for producing AgNPs due to the dual role of biological systems as both reducing and stabilizing agents. Various biological systems, including plants, algae, bacteria, yeast, and fungi, can be used in this process [36].

Plant-mediated synthesis has gained significant attention due to its simplicity, cost-effectiveness, and minimal biohazard risks [37,38]. This method utilizes plant extracts containing natural biomolecules such as polyphenols, flavonoids, and terpenoids, which act as both reducing and stabilizing agents for AgNP formation. Unlike other biological synthesis methods—such as those using bacteria or fungi—plant-mediated synthesis does not require cultivating or maintaining living cell cultures under sterile conditions. This not only simplifies the process but also reduces potential biohazard risks and operational costs, making it a more accessible and environmentally friendly alternative. It further facilitates the formation of capped AgNPs in a one-step reaction, thereby reducing the reliance on multiple chemical reagents. The bacteria-mediated synthesis of highly stable AgNPs has been reported via the bioreduction of aqueous Ag^+^ using the culture supernatant of the nonpathogenic bacterium *Bacillus licheniformis* or enterobacteria [39,40]. *Chlorophyceae*, *Phaeophyceae*, *Cyanophyceae*, and *Rhodophyceae* are the most common types of algae used to synthesize AgNPs [41]. Some fungi-mediated synthesized AgNP advantages are ease of handling, low toxicity of their by-products, and good biocompatibility [42].

The specific mechanisms of AgNP formation through green synthesis can vary depending on factors such as plant extract composition, extraction methods, and reaction conditions, which can influence the size, morphology, and properties of AgNPs, highlighting the need for further research to clarify the detailed mechanisms involved in these systems [43]. Controlling experimental conditions and accurately quantifying phytochemicals, even within the same plant extract, are essential to improve reproducibility and consistently achieve the desired results [44]. This approach will also enhance the yield of AgNPs by reducing time consumption. However, further exploration is needed to understand how varying concentrations of phytochemicals influence the properties of metallic nanoparticles [45].

## 3. Antimicrobial Properties of Silver Nanoparticles

### 3.1. Antibacterial Activity

The remarkable properties of AgNPs make them a promising candidate for use in medical, industrial, and environmental applications. Their unique size-dependent properties, high surface area-to-volume ratio, direct interaction with bacterial cells, and ability to release Ag^+^ contribute to their potent antibacterial activity.

#### 3.1.1. Mechanisms of Antibacterial Activity

Several mechanisms have been proposed to explain the antibacterial effects of AgNPs. One of the primary mechanisms is the oxidative dissolution of AgNPs in aqueous environments, resulting in the formation of highly reactive Ag^+^ that can interact with various cellular components of bacteria, including the cell membrane, proteins, and DNA (deoxyribonucleic acid). This process begins with the oxidation of Ag^0^ to Ag^+^, facilitated by oxidizing agents such as oxygen or H_2_O_2_ in the environment. Silver ions are highly reactive, disrupting bacterial cell wall integrity by binding to thiol groups in proteins and enzymes [46]. This leads to membrane destabilization and the leakage of intracellular contents, such as proteins, nucleic acids, and ions, thereby impairing the bacterial cell’s ability to maintain homeostasis [47]. Using AgNPs, rather than directly applying Ag^+^ solutions such as silver nitrate, offers the advantage of a prolonged and controlled release of Ag^+^ through gradual dissolution, thereby maintaining antimicrobial efficacy over time [48]. The interaction of AgNPs with bacterial cells can result in the generation of ROS, such as H_2_O_2_, superoxide radicals (O_2_^•−^), and hydroxyl radicals (•OH). The production of ROS is considered a key mechanism in the antibacterial action of AgNPs, as ROS are highly toxic to bacterial cells, causing oxidative stress and damaging lipids, proteins, and DNA, which contributes to DNA fragmentation and cell death [49]. The catalytic surface of AgNPs plays a crucial role in facilitating these redox reactions, enhancing ROS production in close proximity to bacterial cells. This process is driven by Fenton-like reactions, resulting in the release of Ag^+^, facilitating the conversion of molecular oxygen into ROS [50]. Under acidic conditions, the H_2_O_2_-mediated oxidation of AgNPs forms reactive intermediates that decompose with protons, generating additional •OH and releasing Ag^+^ [51]. These processes synergistically enhance ROS production and Ag^+^ toxicity, disrupting bacterial cellular redox homeostasis and damaging proteins, lipids, and DNA. This localized generation of ROS further amplifies the antibacterial efficacy of AgNPs.

Furthermore, AgNPs, through their released Ag^+^, have been shown to bind to bacterial DNA, leading to the formation of DNA–Ag^+^ complexes, which interfere with the synthesis of nucleic acids, thus inhibiting bacterial growth and proliferation [52]. In addition, Ag^+^ can interact with and inhibit the activity of critical bacterial enzymes, including those involved in energy production and metabolism, e.g., thiol groups in enzymes like dehydrogenases, potentially disrupting their function and impairing bacterial cellular processes [53]. The antibiofilm activity of AgNPs has been described as dose-dependent [54]. Biofilms are critical for certain bacteria as they provide a protective environment that enhances survival, facilitates nutrient acquisition, and increases resistance to antimicrobial agents and host immune responses [55]. Interestingly, a synergistic effect of AgNPs with antibiotics has been demonstrated in vitro, showing a promising alternative for reducing the quantities of antibiotics used in animals [56]. The proposed synergistic effect is linked to the generation of •OH, disruption of protective cellular functions, and enhanced antibiofilm activity [57].

Lastly, AgNPs have been shown to interfere with bacterial quorum sensing by disrupting the signaling mechanisms that control bacterial communication [58]. They can inhibit the production or reception of autoinducers—the signaling molecules involved in quorum sensing—thereby preventing bacteria from coordinating behaviors, such as biofilm formation, virulence factor expression, and antibiotic resistance [59]. This quorum sensing disruption can weaken bacterial pathogenicity, making AgNPs a potential tool for controlling bacterial infections and reducing the effectiveness of bacterial communication-based mechanisms, particularly in treating topical infections or coating medical implants to prevent bacterial adherence and biofilm development [60].

#### 3.1.2. Factors Affecting Antibacterial Activity

Several factors can influence the antibacterial activity of AgNPs, including their size, shape, surface charge, concentration, and environmental conditions. Smaller AgNPs have a larger surface area-to-volume ratio, which increases their reactivity and the amount of released Ag^+^, thus enhancing their antibacterial activity [61]. Additionally, AgNPs’ shape can affect their interaction with bacterial cells. Spherical nanoparticles are generally more effective than rods or cubes due to a higher surface area, enhanced interaction with bacteria, and better cellular penetration [62,63]. The surface charge of AgNPs influences their interaction with bacterial cell membranes, which are typically negatively charged. Positively charged nanoparticles exhibit more potent antibacterial activity due to enhanced electrostatic attraction to the negatively charged bacterial surface [64]. However, highly positively charged AgNPs could exhibit cytotoxicity to host cells by increasing cellular uptake and causing oxidative stress, inflammation, and cell death [65]. On the other hand, neutrally charged AgNPs also showed high effectiveness against bacteria [64].

**Figure 3:**
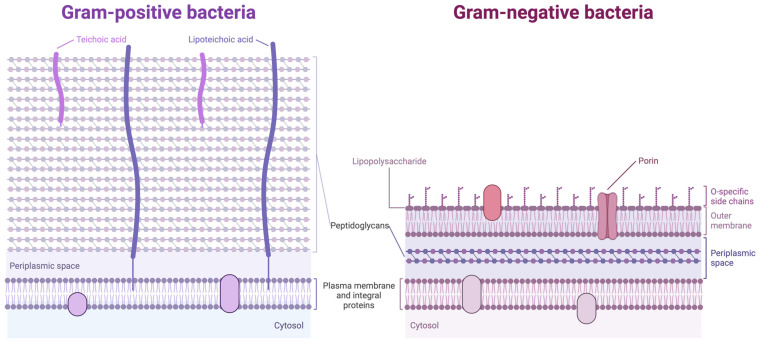
Organization of the cell membrane of Gram-positive and Gram-negative bacteria. Due to their cell membrane peptidoglycan organization, Gram-negative bacteria are more susceptible to silver nanoparticles (AgNPs) than Gram-positive bacteria. Gram-negative bacteria contain lipopolysaccharides in their outer membrane and exhibit only a thin (~3 nm) peptidoglycan layer between the cytoplasmic and outer membranes. In contrast, Gram-positive bacteria lack the outer membrane but have a thicker (~30 nm) peptidoglycan layer, reducing the chances of AgNPs penetration into cells.

The type of bacteria affects their susceptibility to AgNPs. Gram-positive bacteria are less susceptible than Gram-negative bacteria due to the different organization of peptidoglycans within the cell membrane (Figure 3) [66]. Additionally, the negative charge of lipopolysaccharides within the cell membrane of Gram-negative bacteria promotes the adhesion of AgNPs [67,68]. Both classes of bacteria display, however, complete growth inhibition at higher concentrations [69]. Moreover, surface functionalization with various stabilizing agents or biomolecules could modulate the interaction between AgNPs and bacteria, enhancing their biocompatibility or targeting specific bacterial strains [70]. The antibacterial effect is dose-dependent, with higher concentrations of AgNPs generally leading to more potent antimicrobial activity [71]. However, excessively high concentrations could be toxic to host cells or the environment, necessitating careful optimization in therapeutic applications. Moreover, the antibacterial efficacy of AgNPs could be influenced by factors such as pH, temperature, and the presence of organic matter [72].

Finally, the aggregation of AgNPs can decrease their antibacterial effectiveness due to their reduced bacterial attachment, with large particles being more prone to aggregation, and stabilizers are often used to prevent this phenomenon [23,24,30].

#### 3.1.3. Bacterial Resistance to Silver Nanoparticles

Although AgNPs are effective against a wide range of bacteria, concerns have arisen about the potential for bacterial resistance to develop [73,74]. To date, no studies have demonstrated the emergence of such resistance in bacteria isolated from animals. However, prolonged exposure to AgNPs could lead to bacterial phenotypic or genetic changes, either increasing or decreasing bacterial susceptibility to AgNPs or antibiotics, depending on the physicochemical properties of the AgNP formulations [75]. Bacterial resistance mechanisms involve the induction of extracellular AgNP aggregation, the reduction of Ag^+^ to Ag^0^, the prevention of AgNPs from binding and entering cells, and the efflux of AgNPs out of the cells [76].

Furthermore, bacterial biofilms—highly organized communities of bacteria embedded in a protective extracellular matrix—can provide a physical barrier that reduces the penetration of AgNPs, thus contributing to resistance [77]. Within biofilms, bacterial cells are in close proximity, facilitating horizontal gene transfer, such as plasmid exchange, which can promote genetic exchange [78]. This exchange can lead to the development of adaptive resistance to antibiotics, enhancing bacterial survival in the presence of these antimicrobial agents [79]. There are currently no studies on the hazards associated with the spread of resistance mechanisms following the use of AgNPs, representing a significant knowledge gap. Other nanoparticles have been shown to enhance horizontal gene transfer among bacteria [80,81]. More research is needed to better understand bacterial responses to AgNP exposure.

A less commonly recognized fact is that bacterial resistance to antibiotics could also contribute to resistance against AgNPs [74]. Other mechanisms of silver resistance include bacterial mutation, modulation of the surface charge of cell membranes, self-repair of damage, and bacterial motility [76]. Lastly, strategies to mitigate resistance to AgNPs should be applied, such as functionalized AgNPs and minimizing overuse [82,83].

### 3.2. Antiviral Activity

Silver nanoparticles exhibit a range of mechanisms that can inhibit viral replication and infection, making them promising candidates for developing antiviral therapeutics. Their antiviral activity is mediated through several mechanisms interacting with various stages of the viral life cycle, from virus entry into the host cell to replication and assembly. The antiviral activity of AgNPs has been demonstrated against several viruses, including hepatitis B virus, herpesviruses, HIV-1, influenza A virus, and SARS-CoV-2 [84,85,86,87,88].

#### 3.2.1. Mechanisms of Antiviral Activity

One of the most widely recognized mechanisms of AgNPs’ antiviral activity is their ability to adsorb and inactivate viruses. The high surface area-to-volume ratio of AgNPs enables them to interact with viral particles, particularly the viral envelope and capsid. By binding to viral surface proteins, they can inhibit the virus from attaching to and entering host cells. Studies have shown that AgNPs can prevent the attachment of herpes simplex virus, human immunodeficiency virus, and influenza viruses to their respective receptors on host cells [84,89,90]. Moreover, AgNPs can disrupt the lipid bilayer of enveloped viruses, causing structural damage that leads to the disintegration of the viral envelope. This disruption prevents the virus from effectively entering the host cell and initiating infection [91]. Another key mechanism for AgNPs’ antiviral effects is the generation of •OH, H_2_O_2_, and O_2_^•−^ [92]. These highly reactive molecules can cause oxidative damage and impair the integrity of viral particles, leading to the inability to infect host cells and replicate [93]. Silver nanoparticles have also been shown to interfere with viruses’ replication inside host cells. The Ag^+^ released from the AgNPs can interact with the viral RNA (ribonucleic acid) or DNA and disrupt host cellular mechanisms necessary for viral replication—such as enzyme activities and protein synthesis—inhibiting viral genome replication and transcription [87]. In addition to direct antiviral effects, some studies suggest that AgNPs could modulate immune system activity, e.g., by stimulating pro-inflammatory cytokine production [94,95].

#### 3.2.2. Factors Affecting Antiviral Activity

Several factors can influence the antiviral effectiveness of AgNPs, including their size, shape, surface charge, concentration, and the targeted viral strain. Smaller nanoparticles typically exhibit higher surface area-to-volume ratios, which enhances their ability to interact with viral particles [86]. This feature was observed with feline calicivirus, where 10 nm AgNPs exhibited antiviral activity, while 75 nm and 110 nm AgNPs did not. It was suggested that nanoparticle size may influence agglomeration and the dissolution rate of AgNPs [96]. The shape of the nanoparticles could influence their ability to attach to the virus or its components, most of them being spherical [97]. Fewer studies have focused on non-spherical nanoparticles compared to spherical ones, as spherical nanoparticles are believed to play a crucial role in enhancing antiviral effects by facilitating interactions between the nanoparticles and viruses. The surface charge of AgNPs plays an essential role in their interaction with viruses. Positively charged nanoparticles are more likely to interact with the negatively charged components of viral particles—such as the viral capsid or envelope proteins—leading to enhanced antiviral activity, emphasizing the critical role of capping agents [98]. Moreover, the concentration of AgNPs is a crucial determinant of their antiviral activity [97]. At higher concentrations, AgNPs can cause greater disruption to viral particles and more effectively inhibit viral replication [96]. Finally, the susceptibility of different viral strains to AgNPs can vary based on the nature of the virus (e.g., DNA versus RNA viruses) and its structural properties [99]. Enveloped viruses are generally more susceptible to biocidal substances due to their lipid-based membrane, which can be more easily disrupted [100,101].

### 3.3. Antifungal Activity

#### 3.3.1. Mechanisms of Antifungal Activity

Similar to their action against bacteria, AgNPs induce direct physical damage to fungal cell walls and membranes, generation of ROS, interference with cellular processes, and the release of Ag^+^, collectively contributing to the inhibition of fungal growth and the induction of fungal cell death. The interaction of AgNPs with the fungal cell wall leads to structural damage and the weakening of cell integrity [102]. This disruption facilitates the leakage of intracellular contents, leading to cell death. The outer fungal cell wall, composed of chitin, glucans, and proteins, is particularly vulnerable to AgNPs, as these particles can bind to the polysaccharides and proteins, interfering with the cell wall’s structural integrity [103]. The oxidative damage induced by AgNPs is one of the key factors contributing to cell death in some, but not all, fungal species [104]. ROS can cause lipid peroxidation, protein denaturation, and DNA fragmentation, thereby potentially disrupting vital fungal cellular processes [105]. Moreover, Ag^+^ can exert antifungal activity by damaging cellular components such as mitochondria, chromatin, and ribosomes, leading to the inhibition of key metabolic processes [106]. Finally, AgNPs have been shown to interfere with the activity of various fungal enzymes, particularly those involved in cell wall synthesis, protein synthesis, and energy production [107].

#### 3.3.2. Factors Affecting Antifungal Activity

Different factors affect the antifungal activity of AgNPs, including their size, shape, surface charge, concentration, and the type of targeted fungal strain. As observed against bacteria, smaller and positively charged AgNPs exhibit higher antifungal activity than larger and negatively charged ones [108,109]. Higher concentrations of AgNPs typically result in greater antifungal activity, as they can cause more substantial disruption to the fungal cell wall, membrane, and internal structures [110]. The minimum inhibitory concentration of AgNPs can vary depending on the fungal strain and environmental conditions [111].

Similar to bacteria, fungal biofilms are structured communities of fungal cells encased in an extracellular matrix that protect them against environmental stressors, including antifungal drugs. These biofilms significantly reduce the effectiveness of antifungal treatments by limiting drug penetration, enhancing resistance through altered gene expression, and promoting the persistence of fungal infections [112]. However, AgNPs have demonstrated activity against *Candida albicans* biofilm formation by disrupting the outer cell membrane and inhibiting filamentation [113,114].

## 4. Current Clinical Applications of Silver Nanoparticles in Veterinary Medicine

**Figure 4:**
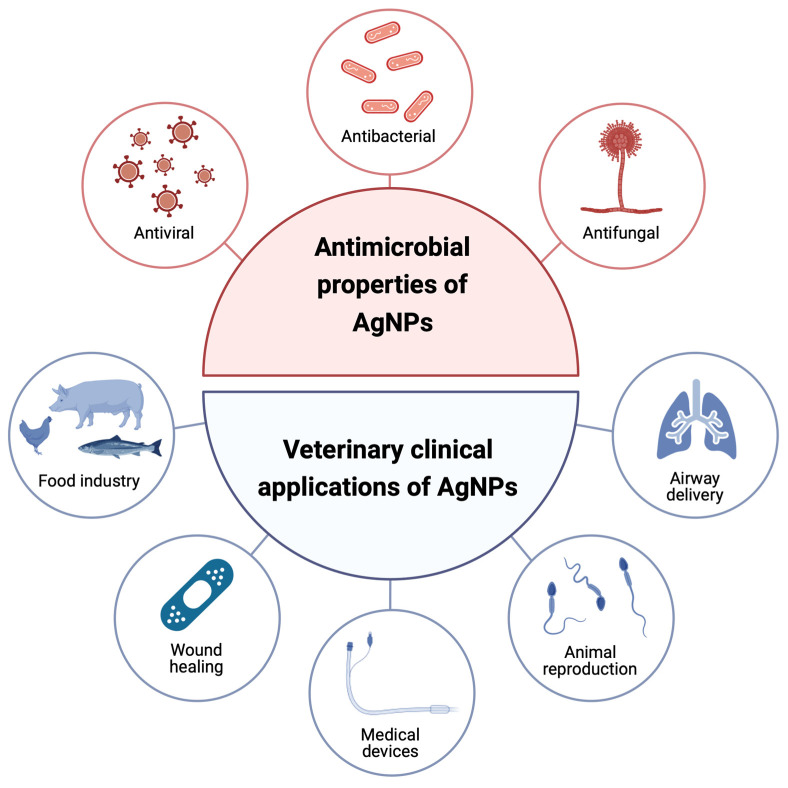
Current clinical applications of silver nanoparticles (AgNPs) in veterinary medicine. As AgNPs exhibit antibacterial, antiviral, and antifungal properties, their use has been investigated for several clinical applications in veterinary medicine.

Due to their antimicrobial properties, the use of AgNPs has been tested in different conditions, including the food industry, wound healing and dressings, medical devices and coatings, animal reproduction, and airway delivery (Figure 4). The applications discussed below highlight the variety of AgNPs used in the referenced studies. These variations include factors such as the form, size, and the presence or absence of a stabilizer, as well as the specific stabilizer employed with the AgNPs. Since size is recognized as a key determinant of antimicrobial activity, it is specified when such information is available. However, the stabilizing agent is not always identified in the referenced studies and, for this reason, is not mentioned.

### 4.1. Ex Vivo Animal Isolate Assays

Several studies have demonstrated the ex vivo efficacy of AgNPs against bacteria. The cell viability and biofilm formation of two multidrug-resistant pathogenic bacteria isolated from uterine secretion samples of dairy cows with postpartum endometritis, *Prevotella melaninogenica* and *Arcanobacterium pyogenes*, were inhibited in a dose- and time-dependent manner [115]. In another design, AgNPs showed antibacterial activity on resistant animal isolates of *Salmonella enterica*, *Staphylococcus aureus*, *Escherichia coli*, *Actinobacillus pleuropneumoniae*, *Streptococcus uberis*, and *Pasteurella multocida* [56]. The antibacterial activity was higher for smaller AgNPs and against Gram-negative bacteria. Tetrahydral AgNPs (~50 nm in size) showed bactericidal properties against clinical isolates of multidrug-resistant *Mycobacterium tuberculosis* and *Mycobacterium bovis*, two pathogens causing tuberculosis, a severe infectious zoonotic disease [116]. Furthermore, AgNPs (size range 10–50 nm) at 12.5 and 25 ppm (parts per million; 1 ppm corresponding to 1 mg/mL) showed antimicrobial properties on pathogens isolated from cows suffering from hoof diseases, including *Sphingoomonas paucimobilis*, *Ochrobactrum* spp., and *Actinomyces odontolyticus* [117]. The antimicrobial activity of AgNPs (~22 nm in size) has been demonstrated against multidrug-resistant strains of *S. enterica* serovar Typhimurium isolated from calves with diarrhea, septicemia, and mortality [118]. Another study showed the antibacterial properties of AgNPs at 10 μg/mL (~10 nm in size) against *S. aureus* isolated from milk samples from cattle with subclinical mastitis within 7 min [119]. A similar observation was made with isolates from milk samples of mastitis-infected goats, where AgNPs (~11 nm in size) demonstrated antibacterial activity in a dose- and time-dependent manner against multidrug-resistant *Pseudomonas aeruginosa* and *S. aureus* [120]. Not insignificantly, AgNPs at concentrations ranging from 0.1 to 2.5 mg/mL (size range 10–50 nm) showed no in vitro toxicity to bovine and human mammary gland cell cultures [121]. The authors concluded that AgNPs could be safe for both cows and humans when used in the milking routine of dairy cows. In poultry, AgNPs (21 nm in size) were effective against two foodborne pathogens, *Salmonella* spp. and *Campylobacter jejuni*, in a dose-dependent manner [122]. The bactericidal effects of AgNPs (size range 11–39 nm) have also been observed against fish pathogens, including *Aeromonas hydrophila* and *Aeromonas salmonicida* [123]. Another study utilizing biosynthesized AgNPs (~4 nm in size) showed antimicrobial activity against marine ornamental fish pathogens *Proteus* spp., *Pseudomonas florescence*, and *Flavobacterium* spp. [124]. The antibiofilm activity of AgNPs (10 nm in size) demonstrated promising results against *Staphylococcus pseudintermedius* isolated from dogs with otitis externa [125]. This bacterium, commonly found as a commensal of canine skin, is known for its antibiotic resistance and is considered a zoonotic agent, posing a particular risk to dog owners [126].

The antiviral activity of AgNPs (~14 nm in size) was demonstrated against the African swine fever virus, a member of the *Asfivirus* genus within the *Asfarviridae* family, which causes high mortality in domestic pigs [127]. At a concentration of 0.78 ppm, AgNPs (14 nm in size) completely inhibited the virus without exhibiting toxicity to porcine alveolar macrophages. Their disinfection ability was also tested in a pig house, where they demonstrated 100% inhibition of *Salmonella* growth when sprayed at a concentration of 25 ppm. The antibacterial efficacy was concentration-dependent, decreasing from 100% to 45% as the AgNP concentration decreased from 25 to 0.025 ppm, respectively. The antiviral activity of AgNPs (~35 nm in size) has also been shown against the Rift Valley fever virus, a zoonotic pathogen affecting domestic animals, including ruminants [128]. Although the ability of AgNPs to control an ongoing infection under the tested conditions was limited, preincubation of the virus with AgNPs before infection in mice significantly reduced infectivity titers and increased the survival rate.

Furthermore, the antifungal activity of AgNPs (~30 nm in size) has been shown on the dermatophyte *Microsporum canis* isolated from dogs and cats [129]. The inhibitory effects of AgNPs (size range 11–39 nm) were also demonstrated against the water mold *Aphanomyces invadans* isolated from infected fish, representing a potential use in novel therapeutics and disease management strategies in aquaculture [123].

### 4.2. Food Industry

Oral administration of AgNPs (4 or 6 mg/kg diet; ~13 nm in size) enhanced performance and significantly decreased both gross and histopathological lesion scores and the expression of virulence genes with experimentally induced *E. coli* colisepticemia—the most common infectious disease of farmed poultry, characterized by the invasion of the bloodstream by coliform bacteria—in broiler chickens [130]. However, dietary supplementation with a higher dose of AgNPs (8 mg/kg diet) resulted in severe adverse effects on all measured parameters (performance, clinical signs, mortality rates, and post-mortem lesions), except gene expression analysis. However, the safety of chicken meat for human consumption was not assessed after treatment with AgNPs.

In another experiment, 30 µg of AgNPs (~15 nm in size) was orally administered to broiler chicks for five successive days post-infection with a pathogenic strain of *Clostridium perfringens*, a bacterium causing necrotic enteritis [131]. One week after infection, the treated chicks showed significantly lower rates of clinical signs (40% versus 15%), mortality (20% versus 3%), and higher body weight gain after two weeks (1124 g versus 1232 g). Macroscopic and histopathological examinations revealed that *C. perfringens* infections caused more severe intestinal lesions in untreated animals than in those treated. Residual silver was detected at a concentration of 413 ng/g in the muscles of treated chicks 16 days after the conclusion of treatment. However, the body weight of 90 broiler chickens supplemented with AgNPs (50 ppm in drinking water; ~3.5 nm in size) was significantly lower than that of control birds, while no differences were observed in the enumeration of bacteria in the contents of cecum, ilium, and feces of the birds [132]. Another study confirmed the lack of effect of AgNPs (20 mg/kg diet; ~27 nm in size) on the cecal microbiota of 870 broilers, while improvements in growth performance (i.e., feed conversion ratio and average daily gain) were observed [133]. The authors found no silver retention in the liver or breast muscle 21 days after the treatment ended.

Furthermore, the positive effects of a commercial AgNP (~18 nm in size) compound on blood parameters have been demonstrated in experimental aflatoxicosis—a fungal toxicosis with typical clinical and pathological symptoms associated with the ingestion of aflatoxins produced by certain molds—in one-day-old broiler chickens during a 28-day experiment [134]. While supplementing AgNPs to a conventional diet had no negative impact on performance, their addition to a diet containing aflatoxin significantly increased both cumulative body weight gain and feed consumption while reducing the feed conversion ratio [135]. The results demonstrate the ability of AgNPs to mitigate the inhibitory effects of aflatoxin.

The potential of AgNPs (size range 2–35 nm) as an antimicrobial growth-promoting supplement for broiler chickens was evaluated by adding them to drinking water [136]. Providing water solutions containing varying concentrations of AgNPs had no significant effect on bacterial populations in intestinal samples, postnatal growth performance, and the energy metabolism of the broiler chickens. However, the administration of AgNPs (~15 nm in size) through drinking water reduced the mortality rate due to *E. coli* and increased feed intake and body weight [137]. At the same time, the risk assessment showed the poultry was fit for human consumption. Furthermore, the in ovo administration of AgNPs (size range 2–35 nm) did not influence embryonic growth but enhanced immunocompetence in chicken embryos [138]. These findings suggest that AgNPs, combined with amino acids, could serve as potential agents to boost both innate and adaptive immunity in chickens. The in ovo preventive and therapeutic actions of AgNPs (~10 nm in size) were observed on infectious bursa disease—also named Gumboro disease—by decreasing the viral growth in embryonated eggs [139].

Oral AgNPs (20 or 40 mg/kg; size range 60–100 nm) were administered for two weeks to weaned pigs [140]. While the ileal concentration of coliforms tended to decrease with treatment, lactobacilli levels remained unaffected. The average daily growth of piglets did not differ between treatments in the first week but showed a linear increase in the second week for animals supplemented with AgNPs. Histological examination of the ileal mucosa revealed no significant changes due to treatment. No severe diarrhea cases were observed during the experiment. Tissue analysis of skeletal muscles and kidneys from pigs fed diets supplemented with AgNPs for five weeks showed no silver accumulation. In contrast, silver was detected in the livers of all AgNP-supplemented animals at an average concentration of 1.35 and 2.45 μg/g dry liver for 20 and 40 mg AgNPs/kg treatments, respectively.

In fish, the in vivo antibacterial activity of AgNPs (100 μg/L; size range 11–39 nm) immersed in water was evaluated against *A. salmonicida* [141]. The treated fish showed no clinical signs, mortalities, or histopathological changes and tested negative for *A. salmonicida*. Additionally, immersion in AgNPs did not result in detectable silver residues in the muscles 35 days post-treatment.

These findings underscore the potential benefits of AgNPs in mitigating disease and enhancing performance in food-producing animals. However, several critical issues remain unresolved. Regulatory challenges and ethical concerns regarding the use of AgNPs in livestock must be addressed, particularly in terms of consumer safety and environmental impacts [142]. Upon oral exposure, AgNPs are absorbed through the gastrointestinal tract, enter the bloodstream, and are distributed to various organs [143]. Prolonged oral administration can lead to organ toxicity and inflammatory responses, with smaller AgNPs showing a tendency to accumulate in organs, such as the brain, lungs, liver, and kidneys [144]. In vivo biodistribution studies have further demonstrated that exposure to AgNPs results in silver accumulation in both local and distant organs [145]. As a site of complex and symbiotic interactions between host cells and the resident microbiota, the gastrointestinal tract plays a vital role in animal health and disease resistance. However, the long-term effects of AgNPs on the gut microbiome remain largely unexplored [146]. Nanosilver can potentially disrupt the microbial balance, potentially leading to clinical conditions such as colitis, obesity, and immune dysfunction [147]. Consequently, the evaluation of AgNPs should not only consider their absorption and accumulation in extraintestinal organs but also their potential effects on gut microbiota and the subsequent implications for the host [143]. The duration of the feeding period emerges as a critical factor in determining the safety of AgNP-based feed additives. From this perspective, feeding animals AgNPs with accumulative tendencies may be safer when limited to short-term applications rather than extended use.

Furthermore, the feasibility and cost-effectiveness of integrating AgNPs into commercial farming operations remain uncertain, as scaling their application could present logistical and economic hurdles. Addressing these issues is essential for determining whether AgNPs can be a viable and sustainable option in modern agriculture.

### 4.3. Wound Healing and Dressings

Nowadays, AgNPs are widely used in wound care products for both humans and animals, including dressings, creams, and gels, for their ability to prevent infection, lower inflammation, and promote wound re-epithelialization [148,149]. They have effectively killed pathogens while being biocompatible and reducing the risk of infection in chronic wounds or burns [150]. An excision wound model in a rabbit showed that an herbal shampoo containing AgNPs (~63 nm in size) decreased the wound area faster than control and with antibiotic streptomycin [151]. In a thermal injury model using male mice, applying bandages coated with AgNPs (14 nm in size) led to reduced inflammation, inhibited bacterial growth, and promoted faster wound healing with less scarring compared to the control group [152]. Additionally, the AgNP treatment influenced the mRNA expression of several cytokines: pro-inflammatory interleukin-6 was downregulated, while anti-inflammatory interleukin-10, pro-inflammatory vascular endothelial growth factor, and pro-inflammatory interferon-gamma were upregulated. Transforming growth factor beta-1 was initially upregulated but later downregulated as the healing progressed. The rate of wound closure was significantly greater in albino rats treated with 10% ointment base containing AgNPs (size range 20–30 nm) compared to those untreated or treated with the topical antibiotic nitrofurazone [153]. Another study also suggested that the same AgNPs have excellent antifungal activity against *C. albicans* and offer a potential antifungal agent for wound healing promotion [154]. A case report showed interest in combining AgNPs (<10 nm in size) and subatmospheric pressure therapy to treat a difficult-to-manage surgical site with progressive wound dehiscence, resistant bacterial infection, and difficulty in applying topical therapy following tumor removal and radiation therapy in a cat [155]. A similar observation was made on a dog with 50% of the total body surface area burned [156]. The combination of AgNPs with vacuum-assisted closure was selected to reduce the frequency of bandage changes to every three days instead of daily, minimizing the need for daily sedation or anesthesia. Finally, AgNP-based dressings promoted enhanced wound healing in donkeys—as evidenced by faster healing, improved cosmetic outcomes, and a stronger antimicrobial effect—in an experimental equine distal limb wound model [157]. Treated wounds exhibited early granulation tissue formation, increased epithelization, and reduced scar width compared to untreated wounds.

When evaluating the use of AgNPs for wound healing, it is essential to strike a careful balance between their cytotoxicity and healing potential. The skin serves as the body’s primary protective barrier, so wounds that compromise this barrier make the body more susceptible to harmful substances [142]. Beyond their specific effects at the wound interface, it is also essential to consider the ability of AgNPs to enter the bloodstream or lymphatic system [158]. Therefore, AgNPs with low cytotoxicity and limited ability to cross animal cell membranes would be more beneficial for wound healing. This highlights the challenges in translating drug development into clinical applications. Additionally, AgNPs have demonstrated superior properties in clearing wound bacteria and reducing healing time compared to traditional treatments like antibiotics, iodine, or chlorhexidine [159,160,161].

### 4.4. Coating of Medical Devices for Veterinary Use

As in human medicine, AgNPs are incorporated into veterinary sutures and medical devices, such as catheters, implants, and surgical instruments, to prevent bacterial colonization and reduce the risk of healthcare-associated infections. Coating polyglactin sutures with AgNPs demonstrated antibacterial efficacy against *S. aureus* and *E. coli*, comparable to antibiotic-coated sutures (i.e., triclosan) [162]. The study revealed that the dual coating of sutures provides a synergistic antibacterial effect. A randomized clinical trial demonstrated the efficacy and safety of AgNP-coated endotracheal tubes in 47 dogs [163]. While no adverse effects or signs of toxicity were observed in the test group, a greater-than-90% reduction in microbial adhesion was noted on the surface of the AgNP-coated tubes compared to uncoated tubes. The use of AgNPs has also been studied on the surface of expandable metallic stents, which are used as implants to treat tracheal stenosis. Covered tracheal stents significantly inhibited granulation tissue formation and collagen deposition in rabbits, ultimately achieving anti-inflammatory and anti-hyperplasia effects [164]. Additionally, covered tracheal stents inhibited bacterial content in the trachea, which could be positively correlated with the reduction in tracheal granulation tissue hyperplasia [165].

Furthermore, coating the outer layer of long bone anastomosis implants in animals with AgNPs demonstrated antibacterial activity against *E. coli* and *S. aureus*, which is beneficial for treating bone tissue [166]. An in vivo study showed that using AgNP-coated implants was safe in six dogs [167]. Moreover, the nanoparticles positively affected the tissue healing process and faster regeneration, suppressing postoperative wound inflammation.

Finally, AgNP-coated screws have been evaluated for the treatment of in situ osteomyelitis—an infective condition of the bone or bone marrow—in animals, both ex vivo in equine cadaver bones and in vivo in osteomyelitic rabbits [168]. Silver-coated pins, particularly at high doses, showed promising results in treating infection in these animal osteomyelitis models without causing toxicity to major organs such as the heart, kidney, and liver at 21- and 42-day time points.

### 4.5. Use in Animal Semen Cryopreservation

A study identified AgNPs (size range 2.7–8.7 nm) as a substitute for antibiotics in bull semen cryopreservation, with good antimicrobial activity, without affecting the motility and fertility of semen [169]. In addition, AgNPs (~2.5 nm in size) exhibited significant antioxidant and antibacterial activity on *E. coli*, *S. aureus*, and *P. aeruginosa* isolated from fresh semen samples of bulls [170]. Antibiotic-free antimicrobial formulations for semen cryopreservation could help prevent the development of bacterial multidrug resistance. However, further research is needed to validate the use of treated semen and assess its impact on the fertilizing ability of spermatozoa. For example, while AgNPs (size range 5–55 nm) showed no harmful effect on the DNA integrity of rabbit sperm assessed by a comet assay, they exhibited significant secondary changes in the morphological structure of spermatozoa [171]. However, the internalization of AgNPs into murine sperm cells inhibited their viability, potentially resulting in impaired fertilization and poor embryonic development [172].

### 4.6. Airway Delivery of Silver Nanoparticles

Given their antibacterial and antiviral properties, the pulmonary delivery of AgNPs may represent a potential treatment for bacterial and viral respiratory infections in animals. Horses are commonly affected by various bacterial and viral pathogens that cause respiratory disorders and may impair performance in equine athletes [173,174]. Respiratory disorders have been identified as the second cause of poor performance in horses, while main equestrian disciplines rely on aerobic metabolism [175,176]. Aerosol therapy is increasingly used in horses to deliver medication directly to the lungs, e.g., to treat equine asthma, a disease characterized by airway inflammation and bronchoconstriction [177].

An in vitro study on the nebulization of AgNPs (~72 nm in size) demonstrated bactericidal activity against two common equine respiratory bacteria, *Streptococcus equi* subsp. *zooepidemicus* and *Actinobacillus equuli* subsp. *equuli* [68]. When AgNPs were instilled, the growth of both bacteria was partially inhibited at a low concentration (100 ppm) and completely inhibited at a high concentration (2000 ppm). However, the nebulization of AgNPs exhibited a differential bactericidal effect on the two bacteria, likely due to the differences in the bacterial structures of Gram-positive and Gram-negative organisms. The growth of Gram-negative *A. equuli* was completely inhibited after the nebulization of AgNPs at a concentration of 100 ppm. In contrast, the growth of Gram-positive *S. zooepidemicus* was only completely inhibited after nebulization with higher concentrations (500, 1000, and 2000 ppm) of AgNPs.

A similar model evaluated the in vitro virucidal activity of the same AgNPs (~72 nm in size) against two viruses [85]. One was the equid alphaherpesvirus-1, a common respiratory virus in horses, and the other was the murine norovirus, which served in this study as a surrogate for tenacious, small, non-enveloped viruses. The nebulization of AgNPs produced distinct virucidal effects on the two viruses, probably due to the presence or absence of an envelope. The enveloped herpesvirus was efficiently inactivated—losing infectivity by at least three orders of magnitude—following both the instillation and nebulization of AgNPs at all tested concentrations (100, 500, 1000, and 2000 ppm). In contrast, while the resistant non-enveloped norovirus was partially inactivated by instillation with AgNPs only at the highest tested concentrations (2000 ppm), it was not inactivated by nebulization of any of the tested concentrations of AgNPs (100, 500, 1000, and 2000 ppm). These results suggest that AgNPs hold potential for antiviral treatment, though their efficacy may vary depending on the virus type.

Until today, in vivo airway delivery of AgNPs has been described only in mice and humans. The intranasal administration of AgNPs (~10 nm in size) in mice significantly improved survival following infection with the H3N2 influenza virus [178]. Mice treated with AgNPs exhibited lower lung viral titers, fewer pathological lesions in lung tissue, and a notable survival advantage during secondary intranasal exposure. The administration of AgNPs (10 nm in size) directly into the trachea of mice before infection with the H3N2 influenza virus or murine pneumonia virus reduced viral loads and, consequently, the levels of virus-induced cytokines [91]. The potential prophylactic antiviral properties of AgNPs were attributed to the recruitment and activation of lymphocytes in the lungs, particularly natural killer cells, and the enhanced ability of alveolar macrophages to promote natural killer cell migration and interferon-gamma production. In contrast, mice treated with AgNPs showed reduced natural killer cell activation following infection, suggesting that AgNPs can regulate the potentially deleterious activation of these cells. Furthermore, the immunomodulatory effects of intranasal AgNPs (10 nm in size) during infection with the respiratory syncytial virus resulted in reduced viral replication and decreased production of pro-inflammatory cytokines in murine lungs [179]. A significant recruitment and activation of neutrophils in the lungs primarily drove the antiviral activity in the airways. Mice inoculated with AgNPs exhibited elevated total protein levels, a marker of epithelial damage and increased vascular permeability. However, they showed no signs of disease, body weight loss, or changes in pulmonary function. Anecdotally, the use of daily mouthwash and nose rinse with AgNPs (~15 nm in size) solutions for nine weeks resulted in a lower incidence of SARS-CoV-2 in health workers during the declaration of the pandemic, with an 84.8% efficiency [180].

Although these studies suggest that AgNPs could serve as a potential antimicrobial treatment for respiratory infections in both animals and humans, the approval of these compounds as formal medical products seems far away at this stage. This is due to the need for further research into their potential toxicity and a better understanding of the mechanisms underlying their antiviral effects. Moreover, after 90 days of inhalation exposure, silver accumulation was more pronounced in the lungs and liver of rats [181]. Silver has also been detected in the brain, olfactory bulb, kidneys, and spleen [181,182,183].

## 5. Potential Adverse Effects of Silver Nanoparticles

Most available information on the toxicity mechanisms and associated effects of AgNPs is derived from in vitro or short-term in vivo studies, with relatively limited data originating from long-term in vivo assessments. Toxicological studies on in vitro animal models have revealed dose- and size-dependent side effects from exposure to AgNPs, including pro-oxidant activity, inflammation, oxidative stress, and changes in mitochondrial distribution. In vitro exposure to AgNPs (25, 40, or 80 nm in size) induced pro-inflammatory responses in rat and porcine brain microvessel endothelial cells, with smaller nanoparticles causing greater cytotoxicity [184,185]. These results suggest that AgNPs could interact with the cerebral microvasculature, potentially leading to brain inflammation and neurotoxicity. Three primary mechanisms of AgNPs’ toxicity have been identified, which are generally dose-dependent: oxidative stress, DNA damage, and cytokine induction [186,187]. A potential mechanism of toxicity involves AgNPs disrupting the mitochondrial respiratory chain, which leads to the production of ROS and interferes with ATP synthesis, ultimately causing DNA damage [186]. This DNA damage is thought to be further exacerbated by the accumulation of AgNPs, which interact with the DNA, leading to cell cycle arrest in the mitotic phase. In vivo studies have shown that exposure to AgNPs can result in adverse effects in various major organs, particularly the liver and bile ducts [142]. Toxicity may depend on the route of administration, with the most studied routes being oral, topical, and inhalation. Notably, in vivo biodistribution studies have shown that exposure to AgNPs leads to the accumulation of silver in both local and distant organs [145]. Following exposure, the clearance behavior of AgNPs plays a crucial role in cumulative toxicity. Several studies have investigated the post-exposure clearance kinetics in rats after subacute inhalation, intravenous, and oral exposure to AgNPs [188,189]. Repeated administration resulted in the accumulation of silver in the liver, lungs, and spleen, suggesting that these organs could be potential targets for toxicity following prolonged exposure [188]. These nanoparticles are typically cleared from most organs within a recovery period ranging from 17 days to 4 months. However, silver persists in long-term oral exposure studies in tissues with biological barriers, such as the brain and testes, indicating the challenges in eliminating it from these organs [189].

The single oral administration of AgNPs (size range 10–20 nm) at a 5000 mg/kg dose did not affect the mice’s hematological parameters, blood chemistry values, or body weight [190]. Furthermore, no gross lesion nor histopathological change was observed in various organs after oral exposure to AgNPs (60 nm in size) until a 1000 mg/kg dose for 28 days [191]. However, doses above 300 mg/kg seemed to induce slight liver damage and the accumulation of silver content in all examined tissues.

The topical application of AgNPs (size range 20–80 nm) for 14 days led to a localized inflammatory response and demonstrated the nanoparticles’ ability to penetrate the surface of porcine skin [192]. Another experiment, using a 24 h topical application of AgNPs (size range 10–20 nm) at concentrations until 100,000 ppm, showed no adverse effects of the nanoparticles related to dermal tissue changes [190]. In rabbits, exposure to 5 mg of AgNPs on a 6 cm^2^ gauze patch resulted in greater dermal toxicity (i.e., erythema and edema) with smaller nanoparticles (10 and 20 nm in size) compared to larger ones (30 nm in size). Furthermore, histopathological findings 14 days after exposure showed dermal changes (e.g., hyperkeratosis, acanthosis, fibrosis, or hyperemia), liver toxicity (e.g., necrosis, mononuclear infiltration, or liver congestion), and splenic and brain toxicity [193].

In studies involving inhalation, rats exposed to AgNPs (18 nm in size) for 6 h per day, 5 days per week over 13 weeks developed dose-dependent changes in lung and liver tissue, including bile-duct hyperplasia, mixed inflammatory cell infiltrates, chronic alveolar inflammation, and small granulomatous lesions [181]. At high concentrations, lung function—measured by tidal volume, minute volume, and peak inspiratory flow—was impaired [194]. Inflammatory responses such as elevated total protein levels, alveolar wall thickening, and macrophage infiltration were also observed in some animals. However, the inhalation of AgNPs (18 nm in size) for 90 days did not appear to induce genetic toxicity in rat bone marrow [195]. A 28-day exposure to inhaled AgNPs (15 nm in size) did not affect body weight, hematological parameters, or blood biochemical values [196]. Moreover, the rapid clearance of AgNPs (15 nm in size) was observed in rats following 6 h inhalation exposure of these nanoparticles [197].

Overall, while significant progress has been made in understanding the toxicological effects of AgNPs, more research is needed to clarify the mechanisms underlying their toxicity following different exposure routes and assess these nanoparticles’ long-term impact on both animal and human health. It is important to highlight that AgNPs may also harm non-target animal organisms (i.e., terrestrial, semi-aquatic, and aquatic species) when released into waste and the environment [198]. Furthermore, AgNPs exert significant toxic activity on marine microalgae, and their effects on plants and soil microorganisms have been described [199,200].

## 6. Future Perspectives of Silver Nanoparticles in Veterinary Medicine

As nanotechnology advances and gains attention, its applications in veterinary medicine are likely to expand in the future. However, replacing antibiotics with AgNPs will likely take longer, as these potential biocidal candidates still require extensive in vivo testing before undergoing clinical trials and food safety assessments in accordance with regulatory standards. The antimicrobial mechanisms of AgNPs remain only partly understood, so further research is needed to elucidate them. Notably, research on the intracellular inhibitory mechanisms of AgNPs is still limited. While oxidative stress induced by AgNPs has received some attention, few studies have explored how AgNPs affect pathogens’ gene expression, protein synthesis, and metabolic processes. These areas warrant further investigation to better understand the full scope of AgNPs’ antimicrobial activity. Although the number of studies on the use of AgNPs in veterinary medicine has increased (Figure 1), further research is necessary to better understand the mechanisms of toxicity associated with different modes of exposure and to define the therapeutic windows in target species.

Furthermore, using AgNPs in veterinary medicine presents regulatory challenges, particularly concerning food safety, residue limits, and environmental impact. Ensuring food safety is a major concern, especially when animals treated with AgNPs are raised for human consumption, as silver may accumulate in animal tissues and contaminate products such as meat, milk, and eggs. Regulatory bodies such as the U.S. Food and Drug Administration and the European Medicines Agency have strict requirements for detecting veterinary drug residues, and establishing safe levels of silver in edible tissues is critical. Setting threshold limits for AgNPs—considering factors such as species, route of exposure, and treatment duration—is necessary to ensure consumer safety. Environmental concerns also arise from the potential accumulation of AgNPs in ecosystems, posing risks to water, soil, plant life, and non-target species, such as aquatic life and soil organisms.

Finally, the potential for pathogen resistance to AgNPs is a growing concern as their use in veterinary medicine grows. Like antibiotics, the overuse or improper use of AgNPs could reduce their effectiveness over time. To mitigate this risk, it is essential to avoid indiscriminate use, establish guidelines for appropriate application, and raise awareness about the dangers of resistance. For example, AgNPs could be reserved for specific indications, and dosing regimens must balance antimicrobial effectiveness and the risk of resistance. Insufficient doses can lead to suboptimal action, fostering resistance. By determining the minimum inhibitory concentration for specific pathogens, veterinary practitioners could ensure more effective use and avoid underdosing. Strategies such as combination therapies and targeted delivery systems can help. Combining AgNPs with other antimicrobial agents could reduce the likelihood of resistance development by promoting synergistic effects (i.e., AgNPs may weaken bacterial cell membranes, making them more susceptible to antibiotics) [201]. Continued research into nanoparticle designs and treatment strategies is essential to maintaining the effectiveness of AgNPs in veterinary applications.

## 7. Conclusions

Silver nanoparticles exhibit strong antibacterial, antiviral, and antifungal activities through various mechanisms, including the release of Ag^+^, membrane and envelope disruption, oxidative stress, inhibition of viral replication, and DNA damage. Their efficacy is influenced by size, shape, surface charge, and concentration, making them highly versatile in combating many pathogens.

Research on AgNPs in the veterinary field is still ongoing, and further studies are needed to clarify their mechanisms of action and assess their long-term effects on animal, human, and environmental health. Nevertheless, their future potential applications in veterinary pharmacology are promising. With continued advancements in nanomedicine, AgNPs have the potential to play a key role in addressing the growing threat of bacterial, viral, and fungal infections in different settings, including veterinary medicine.

## Data Availability

No new data were created or analyzed in this study. Data sharing is not applicable to this article.

## Abbreviations

*A. equuli*, *Actinobacillus equuli* subsp. *equuli*; *A. salmonicida*, *Aeromonas salmonicida*; Ag^+^, silver ion; Ag^0^, elemental silver; AgNPs, silver nanoparticles; *C. albicans*, *Candida albicans*; *C. perfringens*, *Clostridium perfringens*; DNA, deoxyribonucleic acid; *E. coli*, *Escherichia coli*; H_2_O_2_, hydrogen peroxide; O_2_^•−^, superoxide radicals; •OH, hydroxyl radicals; *P. aeruginosa*, *Pseudomonas aeruginosa*; ppm, parts per million; ROS, reactive oxygen species, RNA, ribonucleic acid; *S. aureus*, *Staphylococcus aureus*; *S. zooepidemicus*, *Streptococcus equi* subsp. *zooepidemicus*.

## References

[1] Barillo D.J., Marx D.E. (2014). Silver in Medicine: A Brief History BC 335 to Present. Burns.

[2] Alexander J.W. (2009). History of the Medical Use of Silver. Surg. Infect..

[3] Dangi S., Gupta A., Gupta D.K., Singh S., Parajuli N. (2020). Green Synthesis of Silver Nanoparticles Using Aqueous Root Extract of Berberis Asiatica and Evaluation of Their Antibacterial Activity. Chem. Data Collect..

[4] Ferro Cavalcanti I.M. (2018). Silver Nanoparticles as a Promising Therapeutic Strategy for Infections Caused by Resistant Bacteria in Cattle and Birds. Approaches Poult. Dairy Vet. Sci..

[5] Hill E.K., Li J. (2017). Current and Future Prospects for Nanotechnology in Animal Production. J. Anim. Sci. Biotechnol..

[6] Prabhu S., Poulose E.K. (2012). Silver Nanoparticles: Mechanism of Antimicrobial Action, Synthesis, Medical Applications, and Toxicity Effects. Int. Nano Lett..

[7] Bonilla-Gameros L., Chevallier P., Copes F., Sarkissian A., Mantovani D. (2022). The Oxidation State of Ag Nanoparticles Highly Affects the Release of Ag Ions without Compromising the Mechanical Performance and the Safety of Amorphous Hydrogenated Carbon Coatings. Diam. Relat. Mater..

[8] Ho C.M., Yau S.K.W., Lok C.N., So M.H., Che C.M. (2010). Oxidative Dissolution of Silver Nanoparticles by Biologically Relevant Oxidants: A Kinetic and Mechanistic Study. Chem. Asian J..

[9] Ho C., Wong C., Yau S.K., Lok C., Che C. (2011). Oxidative Dissolution of Silver Nanoparticles by Dioxygen: A Kinetic and Mechanistic Study. Chem. Asian J..

[10] Sharma V.K., Siskova K.M., Zboril R., Gardea-Torresdey J.L. (2014). Organic-Coated Silver Nanoparticles in Biological and Environmental Conditions: Fate, Stability and Toxicity. Adv. Colloid Interface Sci..

[11] Peretyazhko T.S., Zhang Q., Colvin V.L. (2014). Size-Controlled Dissolution of Silver Nanoparticles at Neutral and Acidic PH Conditions: Kinetics and Size Changes. Environ. Sci. Technol..

[12] Mbanga O., Cukrowska E., Gulumian M. (2022). Dissolution Kinetics of Silver Nanoparticles: Behaviour in Simulated Biological Fluids and Synthetic Environmental Media. Toxicol. Rep..

[13] Fernando I., Zhou Y. (2019). Impact of PH on the Stability, Dissolution and Aggregation Kinetics of Silver Nanoparticles. Chemosphere.

[14] Iravani S., Korbekandi H., Mirmohammadi S.V., Zolfaghari B. (2014). Synthesis of Silver Nanoparticles: Chemical, Physical and Biological Methods. Res. Pharm. Sci..

[15] Aryal S., Park H., Leary J.F., Key J. (2019). Top-down Fabrication-Based Nano/Microparticles for Molecular Imaging and Drug Delivery. Int. J. Nanomed..

[16] Xu L., Wang Y.-Y., Huang J., Chen C.-Y., Wang Z.-X., Xie H. (2020). Silver Nanoparticles: Synthesis, Medical Applications and Biosafety. Theranostics.

[17] Thakkar K.N., Mhatre S.S., Parikh R.Y. (2010). Biological Synthesis of Metallic Nanoparticles. Nanomedicine.

[18] Bouafia A., Laouini S.E., Ahmed A.S.A., Soldatov A.V., Algarni H., Feng Chong K., Ali G.A.M. (2021). The Recent Progress on Silver Nanoparticles: Synthesis and Electronic Applications. Nanomaterials.

[19] Khayati G.R., Janghorban K. (2012). The Nanostructure Evolution of Ag Powder Synthesized by High Energy Ball Milling. Adv. Powder Technol..

[20] Elwakil B.H., Eldrieny A.M., Almotairy A.R.Z., El-Khatib M. (2024). Potent Biological Activity of Newly Fabricated Silver Nanoparticles Coated by a Carbon Shell Synthesized by Electrical Arc. Sci. Rep..

[21] Filipović Tričković J., Momčilović M., Joksić G., Živković S., Ilić B., Ognjanović M., Novaković M., Valenta Šobot A. (2023). Laser Ablated Citrate-Stabilized Silver Nanoparticles Display Size and Concentration Dependant Biological Effects. Nanomater. Nanotechnol..

[22] Nur-E-Alam M., Basher M.K., Vasiliev M., Das N. (2021). Physical Vapor-Deposited Silver (Ag)-Based Metal-Dielectric Nanocomposites for Thin-Film and Coating Applications. Appl. Sci..

[23] Bélteky P., Rónavári A., Igaz N., Szerencsés B., Tóth I.Y., Pfeiffer I., Kiricsi M., Kónya Z. (2019). Silver Nanoparticles: Aggregation Behavior in Biorelevant Conditions and Its Impact on Biological Activity. Int. J. Nanomed..

[24] Wu Y., Clark C.J., Lin C., Chen G. (2022). Neutrally Charged Nanosilver Antimicrobial Effects: A Surface Thermodynamic Perspective. Colloids Surf. B Biointerfaces.

[25] Khatoon U.T., Velidandi A., Nageswara Rao G.V.S. (2023). Sodium Borohydride Mediated Synthesis of Nano-Sized Silver Particles: Their Characterization, Anti-Microbial and Cytotoxicity Studies. Mater. Chem. Phys..

[26] Khatoon U.T., Nageswara Rao G.V.S., Mohan K.M., Ramanaviciene A., Ramanavicius A. (2017). Antibacterial and Antifungal Activity of Silver Nanospheres Synthesized by Tri-Sodium Citrate Assisted Chemical Approach. Vacuum.

[27] Pauzi N., Mohamad S., Ghazali S., Jamari S.S. (2023). Evaluation of Glucose Reduction for Silver Nanoparticles Synthesis with Nanocrystalline Cellulose Matrix. Bionanoscience.

[28] Pinheiro L.D.S.M., Sangoi G.G., Vizzotto B.S., Moreno Ruiz Y.P., Galembeck A., Pavoski G., Espinosa D.C.R., Machado A.K., da Silva W.L. (2024). Silver Nanoparticles from Ascorbic Acid: Biosynthesis, Characterization, in Vitro Safety Profile, Antimicrobial Activity and Phytotoxicity. Mater. Chem. Phys..

[29] Augustine R., Hasan A., Thajuddin N., Mathew S. (2020). Multimodal Applications of Phytonanoparticles. Phytonanotechnology.

[30] Amir D., Nasaruddin R.R., Engliman N.S., Sulaiman S., Mastuli M.S. (2021). Effect of Stabilizers in the Synthesis of Silver Nanoparticles and Methylene Blue Oxidation. IOP Conf. Ser. Mater. Sci. Eng..

[31] Gutierrez L., Aubry C., Cornejo M., Croue J.-P. (2015). Citrate-Coated Silver Nanoparticles Interactions with Effluent Organic Matter: Influence of Capping Agent and Solution Conditions. Langmuir.

[32] Vidakis N., Petousis M., Michailidis N., Mountakis N., Papadakis V., Argyros A., Charou C. (2023). Polyethylene Glycol and Polyvinylpyrrolidone Reduction Agents for Medical Grade Polyamide 12/Silver Nanocomposites Development for Material Extrusion 3D Printing: Rheological, Thermomechanical, and Biocidal Performance. React. Funct. Polym..

[33] Knop K., Hoogenboom R., Fischer D., Schubert U.S. (2010). Poly(Ethylene Glycol) in Drug Delivery: Pros and Cons as Well as Potential Alternatives. Angew. Chem. Int. Ed..

[34] Das S., Bandyopadhyay K., Ghosh M.M. (2021). Effect of Stabilizer Concentration on the Size of Silver Nanoparticles Synthesized through Chemical Route. Inorg. Chem. Commun..

[35] Soto-Mendoza A., Larrañaga-Tapia M., Robles E., Martínez-Sanmiguel J.J., Truong L.B., Medina-Cruz D., Cholula-Díaz J.L., Mostafavi E. (2023). Natural Biopolymers Combined with Metallic Nanoparticles: A View of Biocompatibility and Cytotoxicity. Handbook of Natural Polymers.

[36] Duran N., Seabra A.B. (2018). Biogenic Synthesized Ag/Au Nanoparticles: Production, Characterization, and Applications. Curr. Nanosci..

[37] Ahmed S., Ahmad M., Swami B.L., Ikram S. (2016). A Review on Plants Extract Mediated Synthesis of Silver Nanoparticles for Antimicrobial Applications: A Green Expertise. J. Adv. Res..

[38] Song J.Y., Kim B.S. (2009). Rapid Biological Synthesis of Silver Nanoparticles Using Plant Leaf Extracts. Bioprocess. Biosyst. Eng..

[39] Shahverdi A.R., Minaeian S., Shahverdi H.R., Jamalifar H., Nohi A.-A. (2007). Rapid Synthesis of Silver Nanoparticles Using Culture Supernatants of Enterobacteria: A Novel Biological Approach. Process Biochem..

[40] Kalishwaralal K., Deepak V., Ramkumarpandian S., Nellaiah H., Sangiliyandi G. (2008). Extracellular Biosynthesis of Silver Nanoparticles by the Culture Supernatant of Bacillus Licheniformis. Mater. Lett..

[41] LewisOscar F., Vismaya S., Arunkumar M., Thajuddin N., Dhanasekaran D., Nithya C. (2016). Algal Nanoparticles: Synthesis and Biotechnological Potentials. Algae—Organisms for Imminent Biotechnology.

[42] Guilger-Casagrande M., de Lima R. (2019). Synthesis of Silver Nanoparticles Mediated by Fungi: A Review. Front. Bioeng. Biotechnol..

[43] Kazemi S., Hosseingholian A., Gohari S.D., Feirahi F., Moammeri F., Mesbahian G., Moghaddam Z.S., Ren Q. (2023). Recent Advances in Green Synthesized Nanoparticles: From Production to Application. Mater. Today Sustain..

[44] Saleh N., Yousaf Z., Grumezescu A.M. (2018). Tools and Techniques for the Optimized Synthesis, Reproducibility and Scale up of Desired Nanoparticles from Plant Derived Material and Their Role in Pharmaceutical Properties. Nanoscale Fabrication, Optimization, Scale-Up and Biological Aspects of Pharmaceutical Nanotechnology.

[45] Kuppusamy P., Yusoff M.M., Maniam G.P., Govindan N. (2016). Biosynthesis of Metallic Nanoparticles Using Plant Derivatives and Their New Avenues in Pharmacological Applications—An Updated Report. Saudi Pharm. J..

[46] Sondi I., Salopek-Sondi B. (2004). Silver Nanoparticles as Antimicrobial Agent: A Case Study on E. Coli as a Model for Gram-Negative Bacteria. J. Colloid Interface Sci..

[47] Morones J.R., Elechiguerra J.L., Camacho A., Holt K., Kouri J.B., Ramírez J.T., Yacaman M.J. (2005). The Bactericidal Effect of Silver Nanoparticles. Nanotechnology.

[48] Mohamed D.S., Abd El-Baky R.M., Sandle T., Mandour S.A., Ahmed E.F. (2020). Antimicrobial Activity of Silver-Treated Bacteria against Other Multi-Drug Resistant Pathogens in Their Environment. Antibiotics.

[49] Zhang W., Li Y., Niu J., Chen Y. (2013). Photogeneration of Reactive Oxygen Species on Uncoated Silver, Gold, Nickel, and Silicon Nanoparticles and Their Antibacterial Effects. Langmuir.

[50] He D., Miller C.J., Waite T.D. (2014). Fenton-like Zero-Valent Silver Nanoparticle-Mediated Hydroxyl Radical Production. J. Catal..

[51] He W., Zhou Y.-T., Wamer W.G., Boudreau M.D., Yin J.-J. (2012). Mechanisms of the PH Dependent Generation of Hydroxyl Radicals and Oxygen Induced by Ag Nanoparticles. Biomaterials.

[52] Feng Q.L., Wu J., Chen G.Q., Cui F.Z., Kim T.N., Kim J.O. (2000). A Mechanistic Study of the Antibacterial Effect of Silver Ions on *Escherichia coli* and *Staphylococcus aureus*. J. Biomed. Mater. Res..

[53] Holt K.B., Bard A.J. (2005). Interaction of Silver(I) Ions with the Respiratory Chain of *Escherichia coli*: An Electrochemical and Scanning Electrochemical Microscopy Study of the Antimicrobial Mechanism of Micromolar Ag^+^. Biochemistry.

[54] Leng D., Li Y., Zhu J., Liang R., Zhang C., Zhou Y., Li M., Wang Y., Rong D., Wu D. (2020). The Antibiofilm Activity and Mechanism of Nanosilver- and Nanozinc-Incorporated Mesoporous Calcium-Silicate Nanoparticles. Int. J. Nanomed..

[55] Sentenac H., Loyau A., Leflaive J., Schmeller D.S. (2022). The Significance of Biofilms to Human, Animal, Plant and Ecosystem Health. Funct. Ecol..

[56] Smekalova M., Aragon V., Panacek A., Prucek R., Zboril R., Kvitek L. (2016). Enhanced Antibacterial Effect of Antibiotics in Combination with Silver Nanoparticles against Animal Pathogens. Vet. J..

[57] Hwang I., Hwang J.H., Choi H., Kim K.-J., Lee D.G. (2012). Synergistic Effects between Silver Nanoparticles and Antibiotics and the Mechanisms Involved. J. Med. Microbiol..

[58] Saeki E.K., Martins H.M., Camargo L.C.d., Anversa L., Tavares E.R., Yamada-Ogatta S.F., Lioni L.M.Y., Kobayashi R.K.T., Nakazato G. (2022). Effect of Biogenic Silver Nanoparticles on the Quorum-Sensing System of Pseudomonas Aeruginosa PAO1 and PA14. Microorganisms.

[59] Rather M.A., Mandal M. (2023). Attenuation of Biofilm and Quorum Sensing Regulated Virulence Factors of an Opportunistic Pathogen Pseudomonas Aeruginosa by Phytofabricated Silver Nanoparticles. Microb. Pathog..

[60] Qais F.A., Ahmad I., Altaf M., Manoharadas S., Al-Rayes B.F., Ali Abuhasil M.S., Almaroai Y.A. (2021). Biofabricated Silver Nanoparticles Exhibit Broad-Spectrum Antibiofilm and Antiquorum Sensing Activity against Gram-Negative Bacteria. RSC Adv..

[61] Baker C., Pradhan A., Pakstis L., Pochan D.J., Shah S.I. (2005). Synthesis and Antibacterial Properties of Silver Nanoparticles. J. Nanosci. Nanotechnol..

[62] Helmlinger J., Sengstock C., Groß-Heitfeld C., Mayer C., Schildhauer T.A., Köller M., Epple M. (2016). Silver Nanoparticles with Different Size and Shape: Equal Cytotoxicity, but Different Antibacterial Effects. RSC Adv..

[63] Ayala-Núñez N.V., Lara Villegas H.H., del Carmen Ixtepan Turrent L., Rodríguez Padilla C. (2009). Silver Nanoparticles Toxicity and Bactericidal Effect Against Methicillin-Resistant Staphylococcus Aureus: Nanoscale Does Matter. NanoBiotechnology.

[64] Abbaszadegan A., Ghahramani Y., Gholami A., Hemmateenejad B., Dorostkar S., Nabavizadeh M., Sharghi H. (2015). The Effect of Charge at the Surface of Silver Nanoparticles on Antimicrobial Activity against Gram-Positive and Gram-Negative Bacteria: A Preliminary Study. J. Nanomater..

[65] Liao C., Li Y., Tjong S.C. (2019). Bactericidal and Cytotoxic Properties of Silver Nanoparticles. Int. J. Mol. Sci..

[66] Martínez-Castañón G.A., Niño-Martínez N., Martínez-Gutierrez F., Martínez-Mendoza J.R., Ruiz F. (2008). Synthesis and Antibacterial Activity of Silver Nanoparticles with Different Sizes. J. Nanoparticle Res..

[67] Meikle T.G., Dyett B.P., Strachan J.B., White J., Drummond C.J., Conn C.E. (2020). Preparation, Characterization, and Antimicrobial Activity of Cubosome Encapsulated Metal Nanocrystals. ACS Appl. Mater. Interfaces.

[68] Frippiat T., Paindaveine C., Duprez J.-N., Delguste C., Mainil J., Art T. (2021). Evaluation of the Bactericidal Effect of Nebulized Silver Nanoparticles on Common Respiratory Bacteria in Horses– In Vitro Studies. J. Equine Vet. Sci..

[69] Kim J.S., Kuk E., Yu K.N., Kim J.-H., Park S.J., Lee H.J., Kim S.H., Park Y.K., Park Y.H., Hwang C.-Y. (2007). Antimicrobial Effects of Silver Nanoparticles. Nanomedicine.

[70] Ameh T., Zarzosa K., Dickinson J., Braswell W.E., Sayes C.M. (2023). Nanoparticle Surface Stabilizing Agents Influence Antibacterial Action. Front. Microbiol..

[71] Ananda T., Modi A., Managuli V., Mukhopadhyay C. (2023). Antimicrobial Property of Silver Nanoparticles: Effects of Concentration and Temperature on Bacterial Isolates. J. Pure Appl. Microbiol..

[72] Qu F., Xu H., Wei H., Lai W., Xiong Y., Xu F., Aguilar Z.P., Xu H., Wang Y.A. (2010). Effects of PH and Temperature on Antibacterial Activity of Silver Nanoparticles. Proceedings of the 2010 3rd International Conference on Biomedical Engineering and Informatics.

[73] McNeilly O., Mann R., Hamidian M., Gunawan C. (2021). Emerging Concern for Silver Nanoparticle Resistance in Acinetobacter Baumannii and Other Bacteria. Front. Microbiol..

[74] Mann R., Holmes A., McNeilly O., Cavaliere R., Sotiriou G.A., Rice S.A., Gunawan C. (2021). Evolution of Biofilm-Forming Pathogenic Bacteria in the Presence of Nanoparticles and Antibiotic: Adaptation Phenomena and Cross-Resistance. J. Nanobiotechnology.

[75] Kędziora A., Wernecki M., Korzekwa K., Speruda M., Gerasymchuk Y., Łukowiak A., Bugla-Płoskońska G. (2020). Consequences Of Long-Term Bacteria’s Exposure To Silver Nanoformulations With Different Physicochemical Properties. Int. J. Nanomed..

[76] Li H., Xu H. (2024). Mechanisms of Bacterial Resistance to Environmental Silver and Antimicrobial Strategies for Silver: A Review. Environ. Res..

[77] Chien C.-C., Lin B.-C., Wu C.-H. (2013). Biofilm Formation and Heavy Metal Resistance by an Environmental *Pseudomonas* sp.. Biochem. Eng. J..

[78] Madsen J.S., Burmølle M., Hansen L.H., Sørensen S.J. (2012). The Interconnection between Biofilm Formation and Horizontal Gene Transfer. FEMS Immunol. Med. Microbiol..

[79] Michaelis C., Grohmann E. (2023). Horizontal Gene Transfer of Antibiotic Resistance Genes in Biofilms. Antibiotics.

[80] Wang X., Li J., Pan X. (2024). How Micro-/Nano-Plastics Influence the Horizontal Transfer of Antibiotic Resistance Genes—A Review. Sci. Total Environ..

[81] Markowicz A., Borymski S., Adamek A., Sułowicz S. (2023). The Influence of ZnO Nanoparticles on Horizontal Transfer of Resistance Genes in Lab and Soil Conditions. Environ. Res..

[82] Kamat S., Kumari M. (2023). Emergence of Microbial Resistance against Nanoparticles: Mechanisms and Strategies. Front. Microbiol..

[83] Panáček A., Kvítek L., Smékalová M., Večeřová R., Kolář M., Röderová M., Dyčka F., Šebela M., Prucek R., Tomanec O. (2018). Bacterial Resistance to Silver Nanoparticles and How to Overcome It. Nat. Nanotechnol..

[84] Elechiguerra J.L., Burt J.L., Morones J.R., Camacho-Bragado A., Gao X., Lara H.H., Yacaman M.J. (2005). Interaction of Silver Nanoparticles with HIV-1. J. Nanobiotechnology.

[85] Frippiat T., Dams L., Wielick C., Delguste C., Ludwig-Begall L.F., Art T., Thiry E. (2023). In Vitro Virucidal Activity of Nebulized Citrate-Complexed Silver Nanoparticles against Equine Herpesvirus-1 and Murine Norovirus. Virology.

[86] Jeremiah S.S., Miyakawa K., Morita T., Yamaoka Y., Ryo A. (2020). Potent Antiviral Effect of Silver Nanoparticles on SARS-CoV-2. Biochem. Biophys. Res. Commun..

[87] Lu L., Sun R.W.-Y., Chen R., Hui C.-K., Ho C.-M., Luk J.M., Lau G.K., Che C.-M. (2008). Silver Nanoparticles Inhibit Hepatitis B Virus Replication. Antivir. Ther..

[88] Xiang D., Chen Q., Pang L., long Zheng C. (2011). Inhibitory Effects of Silver Nanoparticles on H1N1 Influenza A Virus in Vitro. J. Virol. Methods.

[89] Baram-Pinto D., Shukla S., Perkas N., Gedanken A., Sarid R. (2009). Inhibition of Herpes Simplex Virus Type 1 Infection by Silver Nanoparticles Capped with Mercaptoethane Sulfonate. Bioconjug. Chem..

[90] Papp I., Sieben C., Ludwig K., Roskamp M., Böttcher C., Schlecht S., Herrmann A., Haag R. (2010). Inhibition of Influenza Virus Infection by Multivalent Sialic-Acid-Functionalized Gold Nanoparticles. Small.

[91] Martín-Faivre L., Prince L., Cornu C., Villeret B., Sanchez-Guzman D., Rouzet F., Sallenave J.-M., Garcia-Verdugo I. (2025). Pulmonary Delivery of Silver Nanoparticles Prevents Influenza Infection by Recruiting and Activating Lymphoid Cells. Biomaterials.

[92] Li Y., Lin Z., Zhao M., Xu T., Wang C., Hua L., Wang H., Xia H., Zhu B. (2016). Silver Nanoparticle Based Codelivery of Oseltamivir to Inhibit the Activity of the H1N1 Influenza Virus through ROS-Mediated Signaling Pathways. ACS Appl. Mater. Interfaces.

[93] Kaushik N., Mitra S., Baek E.J., Nguyen L.N., Bhartiya P., Kim J.H., Choi E.H., Kaushik N.K. (2023). The Inactivation and Destruction of Viruses by Reactive Oxygen Species Generated through Physical and Cold Atmospheric Plasma Techniques: Current Status and Perspectives. J. Adv. Res..

[94] Murphy A., Casey A., Byrne G., Chambers G., Howe O. (2016). Silver Nanoparticles Induce Pro-inflammatory Gene Expression and Inflammasome Activation in Human Monocytes. J. Appl. Toxicol..

[95] Shin S.-H., Ye M.-K., Kim H.-S., Kang H.-S. (2007). The Effects of Nano-Silver on the Proliferation and Cytokine Expression by Peripheral Blood Mononuclear Cells. Int. Immunopharmacol..

[96] Bekele A.Z., Gokulan K., Williams K.M., Khare S. (2016). Dose and Size-Dependent Antiviral Effects of Silver Nanoparticles on Feline Calicivirus, a Human Norovirus Surrogate. Foodborne Pathog. Dis..

[97] Luceri A., Francese R., Lembo D., Ferraris M., Balagna C. (2023). Silver Nanoparticles: Review of Antiviral Properties, Mechanism of Action and Applications. Microorganisms.

[98] Sinclair T.R., van den Hengel S.K., Raza B.G., Rutjes S.A., de Roda Husman A.M., Peijnenburg W.J.G.M., Roesink H.D.W., de Vos W.M. (2021). Surface Chemistry-Dependent Antiviral Activity of Silver Nanoparticles. Nanotechnology.

[99] Chen Y.N., Hsueh Y.H., Hsieh C.T., Tzou D.Y., Chang P.L. (2016). Antiviral Activity of Graphene–Silver Nanocomposites against Non-Enveloped and Enveloped Viruses. Int. J. Environ. Res. Public Health.

[100] Wanguyun A.P., Oishi W., Sano D. (2024). Sensitivity Evaluation of Enveloped and Non-Enveloped Viruses to Ethanol Using Machine Learning: A Systematic Review. Food Environ. Virol..

[101] Rakowska P.D., Tiddia M., Faruqui N., Bankier C., Pei Y., Pollard A.J., Zhang J., Gilmore I.S. (2021). Antiviral Surfaces and Coatings and Their Mechanisms of Action. Commun. Mater..

[102] Jian Y., Chen X., Ahmed T., Shang Q., Zhang S., Ma Z., Yin Y. (2022). Toxicity and Action Mechanisms of Silver Nanoparticles against the Mycotoxin-Producing Fungus Fusarium Graminearum. J. Adv. Res..

[103] Li L., Pan H., Deng L., Qian G., Wang Z., Li W., Zhong C. (2022). The Antifungal Activity and Mechanism of Silver Nanoparticles against Four Pathogens Causing Kiwifruit Post-Harvest Rot. Front. Microbiol..

[104] Lee B., Lee M.J., Yun S.J., Kim K., Choi I.-H., Park S. (2019). Silver Nanoparticles Induce Reactive Oxygen Species-Mediated Cell Cycle Delay and Synergistic Cytotoxicity with 3-Bromopyruvate in *Candida albicans*, but Not in *Saccharomyces cerevisiae*. Int. J. Nanomed..

[105] Kumari M., Giri V.P., Pandey S., Kumar M., Katiyar R., Nautiyal C.S., Mishra A. (2019). An Insight into the Mechanism of Antifungal Activity of Biogenic Nanoparticles than Their Chemical Counterparts. Pestic. Biochem. Physiol..

[106] Xia Z.K., Ma Q.H., Li S.Y., Zhang D.Q., Cong L., Tian Y.L., Yang R.Y. (2016). The Antifungal Effect of Silver Nanoparticles on Trichosporon Asahii. J. Microbiol. Immunol. Infect..

[107] Mansoor S., Zahoor I., Baba T.R., Padder S.A., Bhat Z.A., Koul A.M., Jiang L. (2021). Fabrication of Silver Nanoparticles Against Fungal Pathogens. Front. Nanotechnol..

[108] Matras E., Gorczyca A., Przemieniecki S.W., Oćwieja M. (2022). Surface Properties-Dependent Antifungal Activity of Silver Nanoparticles. Sci. Rep..

[109] Akpinar I., Unal M., Sar T. (2021). Potential Antifungal Effects of Silver Nanoparticles (AgNPs) of Different Sizes against Phytopathogenic Fusarium Oxysporum f. Sp. Radicis-Lycopersici (FORL) Strains. SN Appl. Sci..

[110] Kim S.W., Jung J.H., Lamsal K., Kim Y.S., Min J.S., Lee Y.S. (2012). Antifungal Effects of Silver Nanoparticles (AgNPs) against Various Plant Pathogenic Fungi. Mycobiology.

[111] Ribeiro L.G., Roque G.S.C., Conrado R., De Souza A.O. (2023). Antifungal Activity of Mycogenic Silver Nanoparticles on Clinical Yeasts and Phytopathogens. Antibiotics.

[112] Kaur J., Nobile C.J. (2023). Antifungal Drug-Resistance Mechanisms in Candida Biofilms. Curr. Opin. Microbiol..

[113] Ahamad I., Bano F., Anwer R., Srivastava P., Kumar R., Fatma T. (2022). Antibiofilm Activities of Biogenic Silver Nanoparticles Against Candida Albicans. Front. Microbiol..

[114] Lara H.H., Romero-Urbina D.G., Pierce C., Lopez-Ribot J.L., Arellano-Jiménez M.J., Jose-Yacaman M. (2015). Effect of Silver Nanoparticles on Candida Albicans Biofilms: An Ultrastructural Study. J. Nanobiotechnology.

[115] Gurunathan S., Choi Y.-J., Kim J.-H. (2018). Antibacterial Efficacy of Silver Nanoparticles on Endometritis Caused by Prevotella Melaninogenica and Arcanobacterum Pyogenes in Dairy Cattle. Int. J. Mol. Sci..

[116] Selim A., Elhaig M.M., Taha S.A., Nasr E.A. (2018). Antibacterial Activity of Silver Nanoparticles against Field and Reference Strains of Mycobacterium Tuberculosis, Mycobacterium Bovis and Multiple-Drug-Resistant Tuberculosis Strains. Rev. Sci. Et Tech. De L’oie.

[117] Kot M., Kalińska A., Jaworski S., Wierzbicki M., Smulski S., Gołębiewski M. (2023). In Vitro Studies of Nanoparticles as a Potentially New Antimicrobial Agent for the Prevention and Treatment of Lameness and Digital Dermatitis in Cattle. Int. J. Mol. Sci..

[118] Estevez M.B., Casaux M.L., Fraga M., Faccio R., Alborés S. (2021). Biogenic Silver Nanoparticles as a Strategy in the Fight Against Multi-Resistant Salmonella Enterica Isolated from Dairy Calves. Front. Bioeng. Biotechnol..

[119] Dehkordi S.H., Hosseinpour F., Kahrizangi A.E. (2011). An in Vitro Evaluation of Antibacterial Effect of Silver Nanoparticles on Staphylococcus Aureus Isolated from Bovine Subclinical Mastitis. Afr. J. Biotechnol..

[120] Yuan Y.-G., Peng Q.-L., Gurunathan S. (2017). Effects of Silver Nanoparticles on Multiple Drug-Resistant Strains of Staphylococcus Aureus and Pseudomonas Aeruginosa from Mastitis-Infected Goats: An Alternative Approach for Antimicrobial Therapy. Int. J. Mol. Sci..

[121] Kalińska A., Jaworski S., Wierzbicki M., Gołębiewski M. (2019). Silver and Copper Nanoparticles—An Alternative in Future Mastitis Treatment and Prevention?. Int. J. Mol. Sci..

[122] Duffy L.L., Osmond-McLeod M.J., Judy J., King T. (2018). Investigation into the Antibacterial Activity of Silver, Zinc Oxide and Copper Oxide Nanoparticles against Poultry-Relevant Isolates of Salmonella and Campylobacter. Food Control.

[123] Shaalan M.I., El-Mahdy M.M., Theiner S., El-Matbouli M., Saleh M. (2017). In Vitro Assessment of the Antimicrobial Activity of Silver and Zinc Oxide Nanoparticles against Fish Pathogens. Acta Vet. Scand..

[124] Umashankari J., Inbakandan D., Ajithkumar T.T., Balasubramanian T. (2012). Mangrove Plant, Rhizophora Mucronata (Lamk, 1804) Mediated One Pot Green Synthesis of Silver Nanoparticles and Its Antibacterial Activity against Aquatic Pathogens. Aquat. Biosyst..

[125] Seo M., Oh T., Bae S. (2021). Antibiofilm Activity of Silver Nanoparticles against Biofilm Forming *Staphylococcus pseudintermedius* Isolated from Dogs with Otitis Externa. Vet. Med. Sci..

[126] Meroni G., Soares Filipe J.F., Drago L., Martino P.A. (2019). Investigation on Antibiotic-Resistance, Biofilm Formation and Virulence Factors in Multi Drug Resistant and Non Multi Drug Resistant Staphylococcus Pseudintermedius. Microorganisms.

[127] Thi Ngoc Dung T., Nang Nam V., Thi Nhan T., Ngoc T.T.B., Minh L.Q., Nga B.T.T., Phan Le V., Viet Quang D. (2020). Silver Nanoparticles as Potential Antiviral Agents against African Swine Fever Virus. Mater. Res. Express..

[128] Borrego B., Lorenzo G., Mota-Morales J.D., Almanza-Reyes H., Mateos F., López-Gil E., de la Losa N., Burmistrov V.A., Pestryakov A.N., Brun A. (2016). Potential Application of Silver Nanoparticles to Control the Infectivity of Rift Valley Fever Virus in Vitro and in Vivo. Nanomedicine.

[129] Emam M., Soliman M.M.H., Eisa W.H., Hasanin M. (2022). Solid and Liquid Green Ag Nanoparticles Based on Banana Peel Extract as an Eco-friendly Remedy for Ringworm in Pets. Surf. Interface Anal..

[130] Awaad M.H.H., Moustafa K.M.E., Zoulfakar S.A., Elhalawany M.S., Mohammed F.F., El-Refay R.M., Morsy E.A. (2021). The Role of Silver Nanoparticles in the Reluctance of Colisepticemia in Broiler Chickens. J. Appl. Poult. Res..

[131] Salem H.M., Ismael E., Shaalan M. (2021). Evaluation of the Effects of Silver Nanoparticles Against Experimentally Induced Necrotic Enteritis in Broiler Chickens. Int. J. Nanomed..

[132] Vadalasetty K.P., Lauridsen C., Engberg R.M., Vadalasetty R., Kutwin M., Chwalibog A., Sawosz E. (2018). Influence of Silver Nanoparticles on Growth and Health of Broiler Chickens after Infection with Campylobacter Jejuni. BMC Vet. Res..

[133] Zaoui Y., Belanche A., Ben-Jeddou K., Jiménez M.S., Fondevila G., Fondevila M. (2024). Effect of the Dietary Administration Pattern of Silver Nanoparticles on Growth Performance, Biodiversity of Digestive Microbiota and Tissue Retention in Broiler Chickens. Anim. Feed Sci. Technol..

[134] Gholami-Ahangaran M., Zia-Jahromi N. (2014). Effect of Nanosilver on Blood Parameters in Chickens Having Aflatoxicosis. Toxicol. Ind. Health.

[135] Gholami-Ahangaran M., Zia-Jahromi N. (2013). Nanosilver Effects on Growth Parameters in Experimental Aflatoxicosis in Broiler Chickens. Toxicol. Ind. Health.

[136] Pineda L., Chwalibog A., Sawosz E., Lauridsen C., Engberg R., Elnif J., Hotowy A., Sawosz F., Gao Y., Ali A. (2012). Effect of Silver Nanoparticles on Growth Performance, Metabolism and Microbial Profile of Broiler Chickens. Arch. Anim. Nutr..

[137] Kumar I., Bhattacharya J. (2019). Assessment of the Role of Silver Nanoparticles in Reducing Poultry Mortality, Risk and Economic Benefits. Appl. Nanosci..

[138] Bhanja S., Hotowy A., Mehra M., Sawosz E., Pineda L., Vadalasetty K., Kurantowicz N., Chwalibog A. (2015). In Ovo Administration of Silver Nanoparticles and/or Amino Acids Influence Metabolism and Immune Gene Expression in Chicken Embryos. Int. J. Mol. Sci..

[139] Pangestika R., Ernawati R. (2017). Antiviral Activity Effect of Silver Nanoparticles (Agnps) Solution Against the Growth of Infectious Bursal Disease Virus on Embryonated Chicken Eggs with Elisa Test. KnE Life Sci..

[140] Fondevila M., Herrer R., Casallas M.C., Abecia L., Ducha J.J. (2009). Silver Nanoparticles as a Potential Antimicrobial Additive for Weaned Pigs. Anim. Feed Sci. Technol..

[141] Shaalan M., El-Mahdy M., Theiner S., Dinhopl N., El-Matbouli M., Saleh M. (2018). Silver Nanoparticles: Their Role as Antibacterial Agent against Aeromonas Salmonicida Subsp. Salmonicida in Rainbow Trout (*Oncorhynchus mykiss*). Res. Vet. Sci..

[142] Ferdous Z., Nemmar A. (2020). Health Impact of Silver Nanoparticles: A Review of the Biodistribution and Toxicity Following Various Routes of Exposure. Int. J. Mol. Sci..

[143] Bergin I.L., Witzmann F.A. (2013). Nanoparticle Toxicity by the Gastrointestinal Route: Evidence and Knowledge Gaps. Int. J. Biomed. Nanosci. Nanotechnol..

[144] Park E.-J., Bae E., Yi J., Kim Y., Choi K., Lee S.H., Yoon J., Lee B.C., Park K. (2010). Repeated-Dose Toxicity and Inflammatory Responses in Mice by Oral Administration of Silver Nanoparticles. Environ. Toxicol. Pharmacol..

[145] Boudreau M.D., Imam M.S., Paredes A.M., Bryant M.S., Cunningham C.K., Felton R.P., Jones M.Y., Davis K.J., Olson G.R. (2016). Differential Effects of Silver Nanoparticles and Silver Ions on Tissue Accumulation, Distribution, and Toxicity in the Sprague Dawley Rat Following Daily Oral Gavage Administration for 13 Weeks. Toxicol. Sci..

[146] Dahiya D.K., Renuka, Puniya A.K. (2018). Impact of Nanosilver on Gut Microbiota: A Vulnerable Link. Future Microbiol..

[147] Pietroiusti A., Magrini A., Campagnolo L. (2016). New Frontiers in Nanotoxicology: Gut Microbiota/Microbiome-Mediated Effects of Engineered Nanomaterials. Toxicol. Appl. Pharmacol..

[148] Haidari H., Garg S., Vasilev K., Kopecki Z., Cowin A. (2020). Silver-Based Wound Dressings: Current Issues and Future Developments for Treating Bacterial Infections. Wound Pract. Res..

[149] Kalantari K., Mostafavi E., Afifi A.M., Izadiyan Z., Jahangirian H., Rafiee-Moghaddam R., Webster T.J. (2020). Wound Dressings Functionalized with Silver Nanoparticles: Promises and Pitfalls. Nanoscale.

[150] Bold B.-E., Urnukhsaikhan E., Mishig-Ochir T. (2022). Biosynthesis of Silver Nanoparticles with Antibacterial, Antioxidant, Anti-Inflammatory Properties and Their Burn Wound Healing Efficacy. Front. Chem..

[151] Bansod S.D., Bawaskar M.S., Gade A.K., Rai M.K. (2015). Development of Shampoo, Soap and Ointment Formulated by Green Synthesised Silver Nanoparticles Functionalised with Antimicrobial Plants Oils in Veterinary Dermatology: Treatment and Prevention Strategies. IET Nanobiotechnol..

[152] Tian J., Wong K.K.Y., Ho C., Lok C., Yu W., Che C., Chiu J., Tam P.K.H. (2007). Topical Delivery of Silver Nanoparticles Promotes Wound Healing. ChemMedChem.

[153] Gong C.-P., Li S.-C., Wang R.-Y. (2018). Development of Biosynthesized Silver Nanoparticles Based Formulation for Treating Wounds during Nursing Care in Hospitals. J. Photochem. Photobiol. B.

[154] Zhou L., Zhao X., Li M., Lu Y., Ai C., Jiang C., Liu Y., Pan Z., Shi J. (2021). Antifungal Activity of Silver Nanoparticles Synthesized by Iturin against Candida Albicans in Vitro and in Vivo. Appl. Microbiol. Biotechnol..

[155] Woods S., de Castro Marques A.I., Renwick M.G., Argyle S.A., Yool D.A. (2012). Nanocrystalline Silver Dressing and Subatmospheric Pressure Therapy Following Neoadjuvant Radiation Therapy and Surgical Excision of a Feline Injection Site Sarcoma. J. Feline Med. Surg..

[156] Mullally C., Carey K., Seshadri R. (2010). Case Report: Use of a Nanocrystalline Silver Dressing and Vacuum-assisted Closure in a Severely Burned Dog. J. Vet. Emerg. Crit. Care.

[157] Khafaga A.F., Abu-Ahmed H.M., El-Khamary A.N., Elmehasseb I.M., Shaheen H.M. (2018). Enhancement of Equid Distal Limb Wounds Healing by Topical Application of Silver Nanoparticles. J. Equine Vet. Sci..

[158] Hadrup N., Sharma A.K., Loeschner K. (2018). Toxicity of Silver Ions, Metallic Silver, and Silver Nanoparticle Materials after in Vivo Dermal and Mucosal Surface Exposure: A Review. Regul. Toxicol. Pharmacol..

[159] Pathi B.K., Mishra S., Moharana N., Kanungo A., Mishra A., Sahu S., Dash R.K., Dubey R., Das M.K. (2024). Effect of Topical Silver Nanoparticle Formulation on Wound Bacteria Clearance and Healing in Patients with Infected Wounds Compared to Standard Topical Antibiotic Application: A Randomized Open-Label Parallel Clinical Trial. Cureus.

[160] Jiang Y., Zhang Q., Wang H., Välimäki M., Zhou Q., Dai W., Guo J. (2024). Effectiveness of Silver and Iodine Dressings on Wound Healing: A Systematic Review and Meta-Analysis. BMJ Open.

[161] Moaddabi A., Soltani P., Rengo C., Molaei S., Mousavi S.J., Mehdizadeh M., Spagnuolo G. (2022). Comparison of Antimicrobial and Wound-Healing Effects of Silver Nanoparticle and Chlorhexidine Mouthwashes: An in Vivo Study in Rabbits. Odontology.

[162] Pandey V., Gupta A., Choudhary I.S., Imran M., Mudavath S.L., Kar A.G., Nandan R. (2024). Impact of Dual-Coated Silver Nanoparticle and Antibiotic Sutures on Wound Healing in Inflammatory Mouse Models. J. Indian Assoc. Pediatr. Surg..

[163] Lethongkam S., Sunghan J., Wangdee C., Durongphongtorn S., Siri R., Wunnoo S., Paosen S., Voravuthikunchai S.P., Dejyong K., Daengngam C. (2023). Biogenic Nanosilver-Fabricated Endotracheal Tube to Prevent Microbial Colonization in a Veterinary Hospital. Appl. Microbiol. Biotechnol..

[164] Li Z., Tian C., Jiao D., Li J., Li Y., Zhou X., Zhao H., Zhao Y., Han X. (2022). Synergistic Effects of Silver Nanoparticles and Cisplatin in Combating Inflammation and Hyperplasia of Airway Stents. Bioact. Mater..

[165] Li Z., Jiao D., Zhang W., Ren K., Qiu L., Tian C., Li Y., Li J., Zhou X., Zhao Y. (2021). Antibacterial and Antihyperplasia Polylactic Acid/Silver Nanoparticles Nanofiber Membrane-Coated Airway Stent for Tracheal Stenosis. Colloids Surf. B Biointerfaces.

[166] Ziąbka M., Kiszka J., Trenczek-Zając A., Radecka M., Cholewa-Kowalska K., Bissenik I., Kyzioł A., Dziadek M., Niemiec W., Królicka A. (2020). Antibacterial Composite Hybrid Coatings of Veterinary Medical Implants. Mater. Sci. Eng. C.

[167] Ziąbka M., Matysiak K., Cholewa-Kowalska K., Kyzioł A., Królicka A., Sapierzyński R., Januchta-Kurmin M., Bissenik I. (2023). In Vitro and In Vivo Studies of Antibacterial Coatings on Titanium Alloy Implants for Veterinary Application. Int. J. Mol. Sci..

[168] Nandi S.K., Shivaram A., Bose S., Bandyopadhyay A. (2018). Silver Nanoparticle Deposited Implants to Treat Osteomyelitis. J. Biomed. Mater. Res. B Appl. Biomater..

[169] Kanwar A., Virmani M., Lal S., Chaudhary K., Kumar S., Magotra A., Pandey A.K. (2023). Silver Nanoparticle as an Alternate to Antibiotics in Cattle Semen during Cryopreservation. Anim. Reprod..

[170] Yousef M.S., Abdelhamid H.N., Hidalgo M., Fathy R., Gómez-Gascón L., Dorado J. (2021). Antimicrobial Activity of Silver-Carbon Nanoparticles on the Bacterial Flora of Bull Semen. Theriogenology.

[171] Rutkowski M., Grzesiakowska A., Kuchta-Gładysz M., Jarnecka O., Niedbała P., Sękara S., Khachatryan K., Krzemińska-Fiedorowicz L., Khachatryan G. (2024). Alginate Silver Nanoparticles and Their Effect on Sperm Parameters of the Domestic Rabbit. Appl. Sci..

[172] Yoisungnern T., Choi Y.-J., Woong Han J., Kang M.-H., Das J., Gurunathan S., Kwon D.-N., Cho S.-G., Park C., Kyung Chang W. (2015). Internalization of Silver Nanoparticles into Mouse Spermatozoa Results in Poor Fertilization and Compromised Embryo Development. Sci. Rep..

[173] Broux B., Gryspeerdt A., Amory H., Frippiat T., Pardon B., Gasthuys F., Legrand L., Deprez P. (2016). Prevalence of Respiratory Pathogens in Nasal Swabs from Horses with Acute Respiratory Disease in Belgium. Vlaams. Diergeneeskd. Tijdschr..

[174] Frippiat T., van den Wollenberg L., van Erck-Westergren E., van Maanen K., Votion D.-M. (2025). Respiratory Viruses Affecting Health and Performance in Equine Athletes. Virology.

[175] Frippiat T., Votion D.-M. (2024). Warm-Up Strategies and Effects on Performance in Racing Horses and Sport Horses Competing in Olympic Disciplines. Animals.

[176] Frippiat T., van Beckhoven C., van Gasselt V.J., Dugdale A., Vandeweerd J.M. (2023). Effect of Gait on, and Repeatability of Heart Rate and Heart Rate Variability Measurements in Exercising Warmblood Dressage Horses. Comp. Exerc. Physiol..

[177] Frippiat T., Art T., Tosi I. (2023). Airway Hyperresponsiveness, but Not Bronchoalveolar Inflammatory Cytokines Profiles, Is Modified at the Subclinical Onset of Severe Equine Asthma. Animals.

[178] Xiang D., Zheng C., Zheng Y., Li X., Yin J., O’ Conner M., Marappan M., Miao Y., Xiang B., Duan W. (2013). Inhibition of A/Human/Hubei/3/2005 (H3N2) Influenza Virus Infection by Silver Nanoparticles in Vitro and in Vivo. Int. J. Nanomed..

[179] Morris D., Ansar M., Speshock J., Ivanciuc T., Qu Y., Casola A., Garofalo R. (2019). Antiviral and Immunomodulatory Activity of Silver Nanoparticles in Experimental RSV Infection. Viruses.

[180] Almanza-Reyes H., Moreno S., Plascencia-López I., Alvarado-Vera M., Patrón-Romero L., Borrego B., Reyes-Escamilla A., Valencia-Manzo D., Brun A., Pestryakov A. (2021). Evaluation of Silver Nanoparticles for the Prevention of SARS-CoV-2 Infection in Health Workers: In Vitro and in Vivo. PLoS ONE.

[181] Sung J.H., Ji J.H., Park J.D., Yoon J.U., Kim D.S., Jeon K.S., Song M.Y., Jeong J., Han B.S., Han J.H. (2009). Subchronic Inhalation Toxicity of Silver Nanoparticles. Toxicol. Sci..

[182] Wiemann M., Vennemann A., Blaske F., Sperling M., Karst U. (2017). Silver Nanoparticles in the Lung: Toxic Effects and Focal Accumulation of Silver in Remote Organs. Nanomaterials.

[183] Braakhuis H.M., Gosens I., Krystek P., Boere J.A.F., Cassee F.R., Fokkens P.H.B., Post J.A., van Loveren H., Park M.V.D.Z. (2014). Particle Size Dependent Deposition and Pulmonary Inflammation after Short-Term Inhalation of Silver Nanoparticles. Part Fibre Toxicol..

[184] Trickler W.J., Lantz S.M., Murdock R.C., Schrand A.M., Robinson B.L., Newport G.D., Schlager J.J., Oldenburg S.J., Paule M.G., Slikker W. (2010). Silver Nanoparticle Induced Blood-Brain Barrier Inflammation and Increased Permeability in Primary Rat Brain Microvessel Endothelial Cells. Toxicol. Sci..

[185] Trickler W.J., Lantz-McPeak S.M., Robinson B.L., Paule M.G., Slikker W., Biris A.S., Schlager J.J., Hussain S.M., Kanungo J., Gonzalez C. (2014). Porcine Brain Microvessel Endothelial Cells Show Pro-Inflammatory Response to the Size and Composition of Metallic Nanoparticles. Drug Metab. Rev..

[186] Asharani P.V., Mun G.L.K., Hande M.P., Valiyaveettil S. (2009). Cytotoxicity and Genotoxicity of Silver Nanoparticles in Human Cells. ACS Nano.

[187] Haase A., Rott S., Mantion A., Graf P., Plendl J., Thünemann A.F., Meier W.P., Taubert A., Luch A., Reiser G. (2012). Effects of Silver Nanoparticles on Primary Mixed Neural Cell Cultures: Uptake, Oxidative Stress and Acute Calcium Responses. Toxicol. Sci..

[188] Lankveld D.P.K., Oomen A.G., Krystek P., Neigh A., Troost-de Jong A., Noorlander C.W., Van Eijkeren J.C.H., Geertsma R.E., De Jong W.H. (2010). The Kinetics of the Tissue Distribution of Silver Nanoparticles of Different Sizes. Biomaterials.

[189] Lee J.H., Kim Y.S., Song K.S., Ryu H.R., Sung J.H., Park J.D., Park H.M., Song N.W., Shin B.S., Marshak D. (2013). Biopersistence of Silver Nanoparticles in Tissues from Sprague–Dawley Rats. Part Fibre Toxicol..

[190] Maneewattanapinyo P., Banlunara W., Thammacharoen C., Ekgasit S., Kaewamatawong T. (2011). An Evaluation of Acute Toxicity of Colloidal Silver Nanoparticles. J. Vet. Med. Sci..

[191] Kim Y.S., Kim J.S., Cho H.S., Rha D.S., Kim J.M., Park J.D., Choi B.S., Lim R., Chang H.K., Chung Y.H. (2008). Twenty-Eight-Day Oral Toxicity, Genotoxicity, and Gender-Related Tissue Distribution of Silver Nanoparticles in Sprague-Dawley Rats. Inhal. Toxicol..

[192] Samberg M.E., Oldenburg S.J., Monteiro-Riviere N.A. (2010). Evaluation of Silver Nanoparticle Toxicity in Skin in Vivo and Keratinocytes in Vitro. Environ. Health Perspect..

[193] Koohi M.K., Hejazy M., Asadi F., Asadian P. (2011). Assessment of Dermal Exposure and Histopathologic Changes of Different Sized Nano-Silver in Healthy Adult Rabbits. J. Phys. Conf. Ser..

[194] Sung J.H., Ji J.H., Yoon J.U., Kim D.S., Song M.Y., Jeong J., Han B.S., Han J.H., Chung Y.H., Kim J. (2008). Lung Function Changes in Sprague-Dawley Rats after Prolonged Inhalation Exposure to Silver Nanoparticles. Inhal. Toxicol..

[195] Kim J.S., Sung J.H., Ji J.H., Song K.S., Lee J.H., Kang C.S., Yu I.J. (2011). In Vivo Genotoxicity of Silver Nanoparticles after 90-Day Silver Nanoparticle Inhalation Exposure. Saf. Health Work.

[196] Ji J.H., Jung J.H., Kim S.S., Yoon J.U., Park J.D., Choi B.S., Chung Y.H., Kwon I.H., Jeong J., Han B.S. (2007). Twenty-Eight-Day Inhalation Toxicity Study of Silver Nanoparticles in Sprague-Dawley Rats. Inhal. Toxicol..

[197] Takenaka S., Karg E., Roth C., Schulz H., Ziesenis A., Heinzmann U., Schramel P., Heyder J. (2001). Pulmonary and Systemic Distribution of Inhaled Ultrafine Silver Particles in Rats. Environ. Health Perspect.

[198] Exbrayat J.M., Moudilou E.N., Lapied E. (2015). Harmful Effects of Nanoparticles on Animals. J. Nanotechnol..

[199] Moreno-Garrido I., Pérez S., Blasco J. (2015). Toxicity of Silver and Gold Nanoparticles on Marine Microalgae. Mar. Environ. Res..

[200] Ihtisham M., Noori A., Yadav S., Sarraf M., Kumari P., Brestic M., Imran M., Jiang F., Yan X., Rastogi A. (2021). Silver Nanoparticle’s Toxicological Effects and Phytoremediation. Nanomaterials.

[201] Masadeh M.M., Al-Tal Z., Khanfar M.S., Alzoubi K.H., Sabi S.H., Masadeh M.M. (2024). Synergistic Effect of Silver Nanoparticles with Antibiotics for Eradication of Pathogenic Biofilms. Curr. Pharm. Biotechnol..

